# Loss-of-Function Analysis Reveals Distinct Requirements of the Translation Initiation Factors eIF4E, eIF4E-3, eIF4G and eIF4G2 in *Drosophila* Spermatogenesis

**DOI:** 10.1371/journal.pone.0122519

**Published:** 2015-04-07

**Authors:** Sanjay Ghosh, Paul Lasko

**Affiliations:** Department of Biology, McGill University, Montreal, Quebec, Canada; The John Curtin School of Medical Research, AUSTRALIA

## Abstract

In eukaryotes, post-transcriptional regulation of gene expression has a key role in many cellular and developmental processes. Spermatogenesis involves a complex developmental program that includes changes in cell cycle dynamics and dramatic cellular remodeling. Translational control is critical for spermatogenesis in *Drosophila* as many mRNAs synthesized in the spermatocytes are translated only much later during spermatid differentiation. Testes-specific translation initiation factors eIF4E-3 and eIF4G2 are essential specifically for male fertility. However, details of their roles during different stages of spermatogenesis are unknown, and the role of canonical translation initiation factors in spermatogenesis remains unexplored. In this study, we addressed the functional role of *eIF4E-1*, *eIF4E-3*, *eIF4G* and *eIF4G2* in testes development and formation of mature sperm. Using the UAS-Gal4 system and RNA interference, we systematically knocked down these four genes in different stages of germ cell development, and in the somatic cells. Our results show that *eIF4E-1* function in early germ cells and the surrounding somatic cells is critical for spermatogenesis. Both *eIF4E-1* and *eIF4E-3* are required in spermatocytes for chromosome condensation and cytokinesis during the meiotic stages. Interestingly, we find that *eIF4G* knockdown did not affect male fertility while *eIF4G2* has distinct functions during spermatogenesis; it is required in early germ cells for proper meiotic divisions and spermatid elongation while its abrogation in spermatocytes caused meiotic arrest. Double knockdown of *eIF4G* and *eIF4G2* shows that these proteins act redundantly during the early stages of spermatogenesis. Taken together, our analysis reveals spatio-temporal roles of the canonical and testes-specific translation initiation factors in coordinating developmental programs during spermatogenesis.

## Introduction

In sexually reproducing organisms, germ cells transmit the genetic information from parent to offspring, a process central to species survival. In many animal embryos, germ cells are segregated from the soma early in development. Later, they undergo a complex developmental program to differentiate into highly specialized adult gametes. Genetic regulation in germ cells relies heavily on post-transcriptional mechanisms. In many organisms the oocyte nucleus is transcriptionally silent during meiotic arrest, and while maternally-expressed mRNAs are required to drive early embryogenesis, translation of these mRNAs is silenced until fertilization and egg activation. In developing sperm, nuclei become transcriptionally silent upon condensation, thus translational control mechanisms predominate in the final stages of spermiogenesis [1].

**Fig 1:**
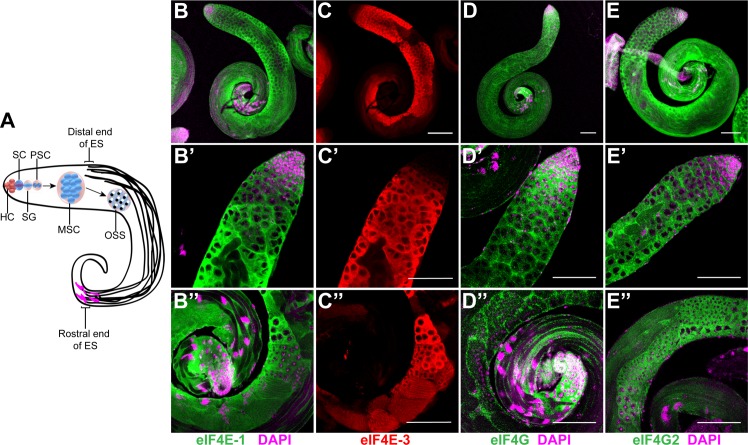
Distribution of eIF4E-1, eIF4E-3, eIF4G and eIF4G2 in the wild-type testes. (A) Schematic diagram showing the stages of spermatogenesis in *Drosophila* testes. The hub cells (HC) at the apical tip of the testes maintain the stem cells (SC) which include a germline stem cell and a somatic cyst stem cell. The germline stem cell differentiates into spermatogonia (SG) which divide mitotically to produce primary spermatocytes (PSC). The spermatocytes undergo rapid cellular growth to form mature spermatocytes (MSC) that undergo meiosis, producing haploid onion stage spermatids (OSS). Following cellular transformation and differentiation, they develop flagella with the nuclei (magenta) at the distal end of the testes. ES = Elongated spermatids. Whole-mount immunostaining of adult testes using antibodies that recognise eIF4E-1 (B-B'', green), eIF4E-3 (C-C'', red), eIF4G (D-D'', green) and eIF4G2 (E-E'', green). The top panel (B-E) shows the entire testes while the middle (B'-E') and lower panels (B''-E'') show the apical and distal end, respectively. DAPI is shown in magenta. Scale bar 100 μm.

Investigations using the fruit fly *Drosophila melanogaster* have provided substantial insight into post-transcriptional mechanisms of genetic regulation in the germ line. In *Drosophila* testes, the successive stages of spermatogenesis are arranged in a linear array (Fig 1A). The apical tip of the testes contains the 'hub' cells, which serve as a niche that maintains the germline stem cell (GSC) and somatic cyst progenitor cell (CPC) populations. The GSC divides mitotically to produce a spermatogonium, which is encapsulated by two cyst cells to generate a cyst. The spermatogonium then undergoes four mitotic divisions with incomplete cytokinesis to generate 16 spermatocytes, which enter an extended G2 phase characterised by a vast increase in cell volume. Following two meiotic divisions, 64 haploid onion-stage spermatids are produced, and each contains a phase-dark mitochondrial aggregate and a phase-light nucleus. Spermiogenesis involves dramatic cellular transformation events that includes formation of the elongated flagellar axoneme structure, nuclear shaping and condensation, and individualisation, to generate the mature sperm with a needle-like nucleus [2,3].

Germ cells in the mitotic and early meiotic stages show abundant transcription, which is shut down at the onset of the first meiotic division [4]. This indicates that mRNAs needed for the meiotic divisions are stored in a translationally repressed state for several days until spermiogenesis [5,6]. Recently, *de novo* RNA synthesis has been reported in the elongating spermatid bundles [7] suggesting that the transcriptional block is not absolute. Indeed, genes that are expressed in the post-meiotic stages have been shown to regulate male fertility [6,8].

Coordination of cell divisions with the ensuing cellular differentiation events is crucial for the formation of mature sperm. Several genes have been identified that are necessary for G2/M transition of meiosis I and onset of spermatid differentiation [9]. However, prior completion of meiosis is not required for activation of the spermatid differentiation program; cysts mutant for one of these genes initiate flagellar elongation, and condensation and shaping of the spermatid nuclei, despite failure to complete meiotic chromosome segregation and cytokinesis [10–12]. Thus, entry into meiosis is sufficient to trigger the differentiation program of male gametogenesis, which points to the existence of a developmental control point at meiosis I.

In eukaryotes, translation initiation is the rate-limiting step of protein synthesis and is a principal target for regulation. In cap-dependent translation, eIF4E binds the 5'-cap structure of mRNAs in the cytoplasm and recruits eIF4G, which through its interaction with eIF4A and PABP leads to recruitment of the 40S ribosomal subunit and circularization of the mRNA [13,14]. The eIF4E-binding protein 4E-BP competitively inhibits the interaction of eIF4G with eIF4E, thus acting as a translation repressor. While components of the eIF4F complex (eIF4E-eIF4G-eIF4A) are conserved across metazoa and play important roles in cellular function, how this complex is regulated has evolved to be different in different organisms and cell types [15]. In *Drosophila*, multiple isoforms of eIF4E and eIF4G exist, and several are expressed primarily in the testes. There are seven *Drosophila* eIF4E proteins, of which eIF4E-1 is the canonical cap-binding protein that is expressed ubiquitously, while eIF4E-3 is testes-specific and essential for male fertility [16,17]. In addition to the canonical eIF4G protein, the eIF4G2 isoform is expressed in testes and is required for fertility in males [18,19]. However, the relationship between the canonical and the testes-specific translation initiation factors has not been fully explored and the function of the testes-specific factors during the different stages of spermatogenesis remains to be determined.

Using loss-of-function analysis, in this study we have systematically examined the function of the translation initiation factors eIF4E-1, eIF4E-3, eIF4G and eIF4G2 in the soma and germline during *Drosophila* testes development. We concentrated on these because eIF4E-1 and eIF4G are canonical translation factors essential for viability, while eIF4E-3 and eIF4G2 have known functions in spermatogenesis. The proteins were knocked down by RNA interference during different stages of spermatogenesis using the UAS-Gal4 system. We find that early expression of eIF4E-1 in the germ cells and the surrounding somatic cyst cells is essential for gonad morphogenesis and germline development. Later, in spermatocytes, eIF4E-1 together with eIF4E-3 regulates chromosome condensation during meiosis and spermatid maturation. We show that eIF4G and eIF4G2 act redundantly in the germ cells during early gametogenesis to control testes development while knockdown of eIF4G in cyst cells affects meiotic stages and differentiation events. Finally, our results demonstrate distinct functions of eIF4G2 in the germ cells during spermatogenesis, as knockdown in the early stages abrogates spermatid elongation, while its function in the spermatocytes is critical for meiotic entry. Taken together, our analysis reveals that germline-soma crosstalk during early spermatogenesis is essential for gonadogenesis, demonstrating distinct functions for the translation initiation factors in regulating progression of germ cell development.

## Materials and Methods

### Fly stocks

Oregon-R flies were used as wild-type controls. For knockdown of genes, transgenic flies generated by the TRiP project (Harvard Medical School) were obtained from Bloomington Drosophila Stock Centre as follows: *eIF4E-1* (BL#34096), *eIF4E-3* (BL#42804), *eIF4G* (BL#33049) and *eIF4G2* (BL#35809). The driver lines were *nos*-Gal4:VP16, *bam*-Gal4:VP16/TM3 (gift of M. Fuller), c587-Gal4:VP16 (gift of M. Fuller), *Ubi*-Gal4 (BL#32551). Other fly stocks were *twine*-*lacZ/CyO* (gift of M. Fuller), *Sa*-GFP (gift of M. Fuller), *CyclinB-GFP* (BL#51568) and *dj-GFP* (BL#5417). All crosses were kept at 25°C except for the knockdown of *eIF4G* with c587 driver, which was carried out at 29°C.

### Microscopy

Testes were dissected in PBS and processed for whole mount immunostaining as described previously [20]. The primary antibodies used were: rabbit anti-eIF4E-1 [21] (1:500), rat anti-eIF4E-3 [17] (1:500), rabbit anti-eIF4G (1:500, raised against peptide QNMILPANKKTKKYDQQVPTSKPQS), rabbit anti-eIF4G2 (1:200, raised against peptide HTDLDTALDDNSTLC), mouse anti-Orb 4H8 (Developmental Studies Hybridoma Bank, 1:25). Goat anti-rabbit, anti-rat and anti-mouse antibodies conjugated with Alexa 488 or Alexa 633 (Life Technologies) were used as secondary antibody. DAPI (Invitrogen #D3571) was used at 10 μg/ml. Fluorescent *in situ* hybridisation was performed as described previously [22]. DIG-labelled sense and antisense probes were prepared using *eIF4E-3* coding region as template by using DIG RNA Labeling kit (Roche #11277073910). After hybridization, the testes were incubated with anti-DIG antibody conjugated with POD (Roche) and developed using TSA Cyanine 3 Tyramide Reagent (PerkinElmer #SAT704A001EA). To monitor GFP fluorescence, testes were fixed in 4% paraformaldehyde for 5 min, washed in 0.1% PBS-Triton X-100 and stained with DAPI for 5 min. β-galactosidase activity assay for Twine-LacZ expression was carried out as in [23]. Phase contrast-Hoescht staining was performed as described in [24].

The images for the immunostaining, *in situ* hybridisation and GFP fluorescence were obtained using Zeiss LSM510 meta confocal laser scanning microscope at the CIAN, Dept. of Biology, McGill University. The Twine-LacZ and live cell phase contrast samples were analyzed in Leica DM6000B microscope. All images were processed by the Fiji software [25].

### Western blotting

For SDS PAGE, protein extract from 3 pairs of testes were loaded into each lane of a 12% gel. The primary antibodies used were: rabbit anti-eIF4E-1 [21] (1:1000), rat anti-eIF4E-3 [17] (1:2000), mouse anti-α-tubulin (Sigma #T6199, 1:20000). Goat anti-Rabbit (1:5000) and anti-rat (1:2500) conjugated with HRP (GE Healthcare) were used as secondary antibodies.

### Co-immunoprecipitation

All procedures were performed at 4°C. Testes from wild-type males (~300) were dissected in PBS, lysed in 1 ml of cold lysis buffer (20 mM HEPES pH 7.5, 150 mM KCl, 4 mM MgCl_2_, 0.1% (v/v) NP-40, 0.5 mM DTT, 1x Halt protease inhibitor) and centrifuged at 13,000 x g for 10 min. The supernatant was pre-cleared with 100 μl protein A agarose beads (Roche) for 30 min and incubated separately with normal guinea pig IgG (Santa Cruz; 1:100), rabbit anti-eIF4G (1:50), rabbit anti-eIF4G2 (1:25) for 2 h on a orbital rotator. 20 μl of BSA-blocked protein A agarose beads were added to the extract which was incubated further for 1 h. Subsequently, the beads were washed with lysis buffer 3 times for 30 min followed by a PBS wash for 5 min. The beads were boiled in SDS sample buffer and the supernatant was used for SDS-PAGE analysis. For western analysis, the primary antibodies were rabbit anti-eIF4G (1:1000), rabbit anti-eIF4G2 (1:200), rat anti-eIF4E-3 (1:2000), rabbit anti-eIF4E-1 (1:1000), rabbit anti-PABP (1:8000), rabbit anti-4E-BP (1:1000). HRP-conjugated goat anti-rabbit (1:5000) and anti-rat (1:2500) antibodies (GE Healthcare) were used as secondary antibodies.

## Results

### Distribution of eIF4E-1, eIF4E-3, eIF4G and eIF4G2 in *Drosophila* testes

In *Drosophila*, eIF4E-1 and eIF4G are canonical translation initiation factors, while eIF4E-3 and eIF4G2 have been reported to be testes-specific [17–19]. To begin to investigate the functions of these translation initiation factors during the different stages of spermatogenesis, we first determined their distribution pattern in detail by immunostaining of wild-type testes. The identity of different cell types in the testes was established using cell type-specific markers, assessing cell morphology, and observing chromatin organization as revealed by DAPI staining. To detect eIF4E-1 and eIF4E-3 simultaneously in the testes, we used antisera that have been shown to recognize each of these proteins specifically [17,21]. Co-staining of the testes with these antisera revealed a cytoplasmic distribution of the proteins (Fig 1B and 1C and S1A–S1A'' Fig and S1D–S1D'' Fig). However, they were expressed in different domains in the testes.

eIF4E-1 was expressed throughout many stages of spermatogenesis, both in the germ cells and surrounding somatic cyst cells (Fig 1B–1B'' and S1D and S1D'' Fig, arrow). The protein was detected in the hub cells containing the stem cell niche at the apical tip of the testes, spermatogonia, primary and secondary spermatocytes, as well as in the early stages of spermatid elongation. Although absent in the mature elongated spermatid bundles undergoing differentiation, the protein was present in the surrounding cyst cells at this stage (Fig 1B'' and S3C Fig). In contrast, eIF4E-3 expression was undetectable at the apical tip of the testes, in the region containing the stem cells and spermatogonia, as reported previously (Fig 1C and 1C', [17]). eIF4E-3 first appeared in the primary spermatocytes, coincident with Sa-GFP expression (S1F' Fig and S1G' Fig), and it was abundantly expressed in the growth phase of the mature spermatocytes and persisted through the meiotic elongating spermatid stages (Fig 1C–1C'' and S1D' and S1D'' Fig).

To determine whether the absence of eIF4E-3 protein from the apical tip of the testes is due to transcriptional or post-transcriptional control, we performed fluorescent *in situ* hybridisation using antisense *eIF4E-3* riboprobe. As shown in S1C–S1C' Fig, the mRNA was expressed in a pattern similar to the corresponding protein, suggesting that expression of eIF4E-3 is primarily regulated at the transcriptional level. In contrast with eIF4E-1, eIF4E-3 was absent from the somatic cyst cells at all stages of spermatogenesis and accumulated exclusively in germ cells (S1D'' Fig). However, the expression domains of eIF4E-1 and eIF4E-3 in the testes overlap considerably from the mature spermatocytes to the elongating spermatid stage (S1A–S1A'' Fig). This raises the possibility that both proteins function in germ cells during meiotic and post-meiotic stages.

We next investigated the distribution patterns of eIF4E-interacting proteins eIF4G and eIF4G2 within the testes. For immunostaining, we used affinity-purified antibodies raised against peptides specific for each protein (see Materials and Methods). We found that eIF4G was expressed primarily in the somatic cyst cells. In the germ cells, some eIF4G staining was detected in the cytoplasm of spermatogonia and primary spermatocytes, while it was clearly excluded from meiotic and post-meiotic stage germ cells (Fig 1D–1D''). In contrast, eIF4G2 accumulated in the cytoplasm of male germ cells as well as in the surrounding somatic cyst cells in all stages of spermatogenesis (Fig 1E–1E''). The protein was abundant in all developmental stages from mature spermatocytes through post-meiotic elongating spermatids, a pattern similar to that of eIF4E-1 and eIF4E-3 (S1E–S1E'' Fig). Notably, we consistently found eIF4G2 staining at the rostral and caudal end of the elongated spermatid bundles.

Taken together, our analysis reveals a distinct distribution pattern for the canonical and testes-specific translation initiation factors; eIF4E-1, eIF4G and eIF4G2 are expressed in both soma and germline, while eIF4E-3 is germline-specific.

### Functional analysis of eIF4E-1, eIF4E-3, eIF4G and eIF4G2 by RNAi

Flies mutant for either *eIF4E-3* or *eIF4G2* are male sterile [17–19], but it is not known whether these proteins are required only in the germ line. In addition, the function of eIF4E-1 and eIF4G in male fertility has not been assessed as they are essential for cell viability and thus mutants in these genes are recessive lethal.

To investigate the function of these translation factors comprehensively during spermatogenesis, we used RNA interference (RNAi) and the UAS-Gal4 system [26] to achieve specific knockdown of gene function in a spatio-temporal manner. Adult males expressing short hairpin RNAs (shRNAs) targeting the gene of interest were generated by crossing transgenic fly lines containing the UAS-shRNA constructs (TRiP collection, [27]) with Gal4 driver lines that are either germline specific (*nos*-Gal4:VP16 and *bam*-Gal4:VP16, [28]) or active in somatic cyst cells (c587-Gal4, [29,30]). We confirmed the expression domains of the germline-specific drivers within the testes by using them to drive a UAS-eGFP fly line. Activity of the *nos* promoter was restricted to the apical tip of the testes, as the GFP signal was primarily enriched in the stem cell niche and spermatogonial cells (S2A and S2B Fig). Importantly, somatic cyst cells did not show any GFP expression. We also detected some GFP signal in the primary spermatocytes, which could be due to slow turnover of the fluorescent protein.

In contrast, when expressed using the *bam*-Gal4:VP16 driver, the GFP signal was excluded from the stem cell niche and possibly also from spermatogonia, at the testes apical tip (S2C Fig and S2D Fig). Early spermatocytes showed abundant GFP expression, which tapered off through the later mitotic stages. GFP signal was not detected in the cyst cells that encapsulate the developing germ cells. This is consistent with the established pattern of endogenous Bam protein in the *Drosophila* testes [31].

### An early function of eIF4E-1 in testes development

**Fig 2:**
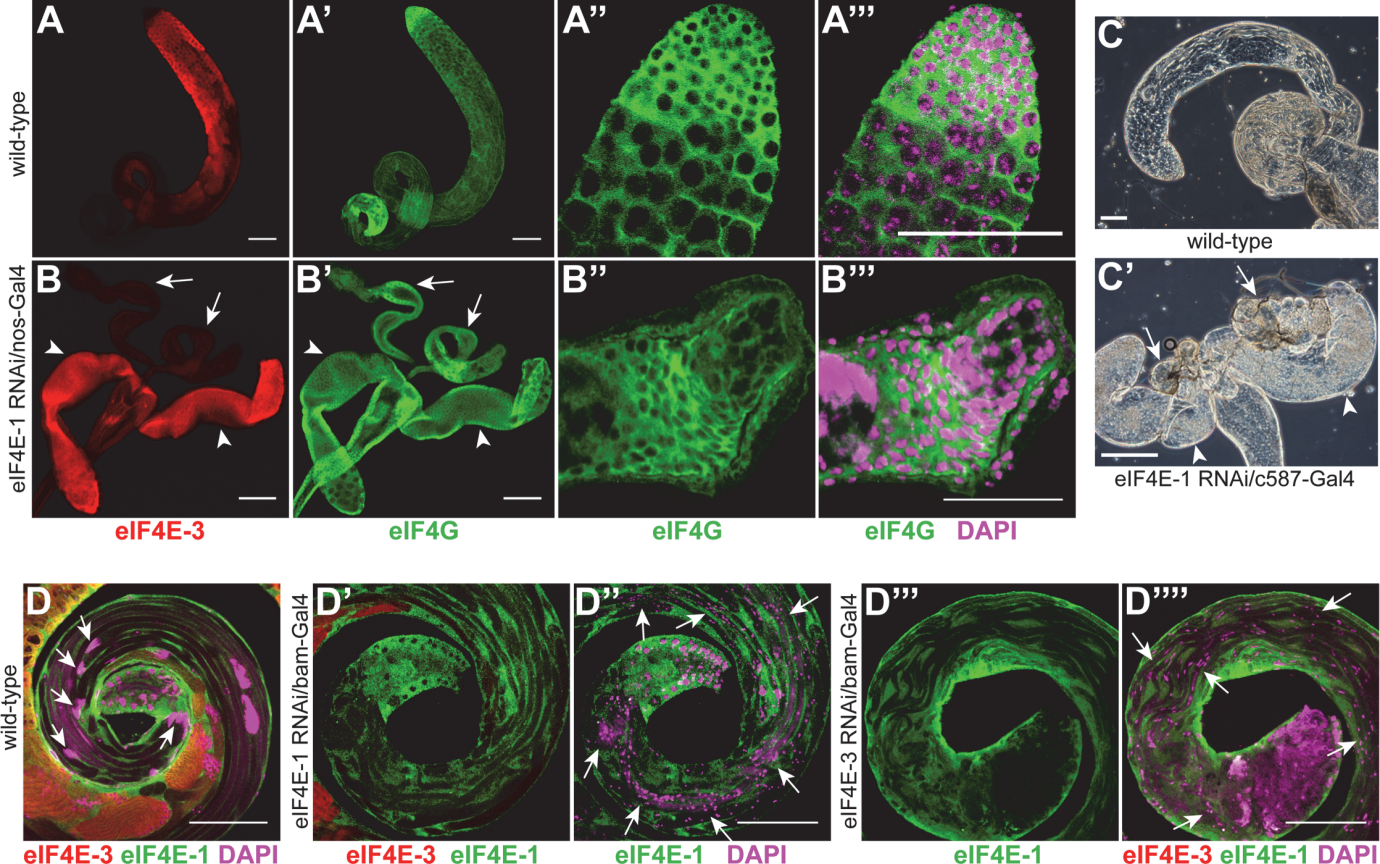
Knockdown of *eIF4E-1* and *eIF4E-3* affects testes development and spermatid differentiation. Wild-type testes (A-A''') and testes expressing *eIF4E-1* shRNA with *nos*-Gal4:VP16 driver (B-B''') were stained with anti-eIF4E-3 (red, A, B) and anti-eIF4G antibody (green, A', A'' & B', B''). The small rudimentary testes are marked by arrows while the accessory glands are marked by arrowheads (B, B'). A magnified view of the apical tip of *eIF4E-1* knockdown testes shows a mass of undifferentiated cells surrounded by a disorganised muscle sheath (B'', B''') as compared with the wild-type (A'', A'''). The DNA is stained with DAPI (magenta). Scale bars 100 μm (A, A', B, B') and 50 μm (A''', B'''). Expression of *eIF4E-1* shRNA using c587-Gal4 driver results in a degenerate testes structure (C', arrows) as revealed by phase contrast microscopy. The arrowheads indicate the accessory glands. The wild-type testis is shown in C. Scale bar 100 μm. (D) The distal end of wild-type testes, stained with anti-eIF4E-1 (green) and anti-eIF4E3 (red) antibody shows well organized haploid nuclear bundles (magenta, arrows). A similar region of a *bam*-Gal4-*eIF4E-1* RNAi testis (D', D'') co-stained with anti-eIF4E-1 (green) and anti-eIF4E-3 (red) antibody reveals severe defects in nuclear compaction and individualisation; the post-meiotic nuclei (stained with DAPI, magenta) are found along the elongated spermatids (arrows). Scale bar 100 μm. (D''', D'''') Knockdown of *eIF4E-3* in spermatocytes using *bam*-Gal4:VP16 driver results in loss of nuclear condensation and bundling at the distal end of the testes (arrows). eIF4E-3 and eIF4E-1 immunostaining is shown in red and green, respectively. The DNA is stained with DAPI (magenta). Scale bar 100 μm.

At the apical tip of the testes, eIF4E-1 but not eIF4E-3 is expressed in the germ cells as well as the surrounding cyst cells (Fig 1B and 1B'). This suggests a role for germline-expressed eIF4E-1 during the early stages of testes development. To assess its function in the stem cell niche and early germ cells, we expressed shRNA targeting *eIF4E-1* using the *nos* driver. Males expressing *eIF4E-1* shRNA under the *nos* promoter were sterile. Their testes were considerably smaller than wild-type, and they did not produce differentiated cell types characteristic of the different stages of spermatogenesis (compare Fig 2A and 2A' with 2B and 2B'). Instead, the apical tip of the testes contained a mass of cells that did not organise into germ cell cysts (Fig 2B'' and 2B'''), while the rest of the structure consists of a lumen that was devoid of germ cells (Fig 2B'). None of the cells in the testes immunoreacted with the anti-eIF4E-3 antibody (Fig 2B), indicating an absence of spermatocytes and spermatids, and further indicating that eIF4E-3 is not upregulated when eIF4E-1 is reduced. DAPI staining of these testes showed a chromosome condensation state that is characteristic of spermatogonial cells (Fig 2B'''). These results indicate that primary spermatocytes do not form in the absence of eIF4E-1, and that germ line development is blocked during or before the transit-amplifying divisions. Notably, the epithelium surrounding the apical cell mass appeared disorganized and was multi-layered (Fig 2B''). Taken together, our data supports a role of eIF4E-1 in early stages of spermatogenesis, showing that it is essential for germ cell cyst formation and germ cell differentiation.

To examine whether eIF4E-1 is required in somatic cells for testes development, we expressed shRNA targeting *eIF4E-1* in cyst cells using c587-Gal4. Males knocked down for *eIF4E-1* in cyst cells were sterile and produced only rudimentary testes (Fig 2C'), which appeared smaller and disorganized than the germline knockdown as described above. This suggests that eIF4E-1 is essential for somatic cell viability, and that germline differentiation does not proceed in the absence of proliferating cyst cells. Finally, we found that knockdown of *eIF4E-3* using either the *nos* or the c587 driver (S9A–S9A'' Fig) did not affect male fertility or testes development, which was unsurprising as endogenous eIF4E-3 is not present in soma or in germ cells that express *nos*.

### eIF4E-1 and eIF4E-3 are essential for meiotic and post-meiotic differentiation

The expression domains of eIF4E-1 and eIF4E-3 overlap extensively in post-mitotic germ cells (S1A–S1A'' Fig), indicating a role of the proteins in these processes. *eIF4E-3* is essential for male fertility and is genetically required for proper execution of the meiotic stages of spermatogenesis [17]. However, the function of *eIF4E-1* and its relationship with *eIF4E-3* during these stages has not been explored. To address this, we generated flies expressing shRNAs targeting either *eIF4E-1* or *eIF4E-3* using the *bam*-Gal4:VP16 driver and investigated its effect on male fertility.

*bam*-Gal4:VP16-mediated knockdown of either *eIF4E-1* or *eIF4E-3* caused sterility. Immunostaining of *eIF4E-1* RNAi testes with anti-eIF4E-1 antibody revealed a severe knockdown of eIF4E-1 in the germ line cysts starting from the primary spermatocyte to the elongating spermatid stages (compare S3A–S3C Fig with S3B–S3D Fig). Nevertheless, knockdown of *eIF4E-1* during these developmental stages did not affect the gross development and morphology of the germ cells or the distribution of eIF4E-3 and eIF4G2 proteins within the testes (S3B' Fig, S3D' Fig, S4B Fig and S4B' Fig). In contrast with wild-type though, in *eIF4E-1* knockdown testes we failed to detect needle-like nuclear bundles at the rostral end of the elongated spermatids; instead, the nuclei were round and distributed along the entire length of the flagellar axoneme cysts (compare Fig 2D with 2D'' and S4A' Fig with S4B' Fig). These observations indicate a failure of nuclear condensation and individualization during spermatid differentiation when eIF4E-1 is abrogated in later stages of spermatogenesis.

To examine whether these phenotypes correlate with defects in the cell division program, we examined the expression and distribution of the cell cycle regulators Twine and Cyclin B (CycB) in testes expressing shRNA targeting *eIF4E-1* driven by *bam*-Gal4:VP16. In *Drosophila*, removal of the inhibitory phosphorylation of Cdc2 by the Cdc25 phosphatase Twine is necessary to initiate the G2/M transition of meiosis I [10,32]. In wild-type testis, Twine, as revealed by the Twine-LacZ translational reporter, accumulates in mature spermatocytes (S5A'' Fig). Knockdown of *eIF4E-1* did not significantly alter the Twine-LacZ expression pattern, indicating that mature primary spermatocytes can initiate meiosis I division in the absence of eIF4E-1 (S5B'' Fig). CycB, on the other hand, shows a dynamic distribution during spermatogenesis; it is enriched in the mitotic region at the apical tip of the wild-type testis and later in the cytoplasm of mature primary spermatocytes (S5A Fig and S5A' Fig). CycB is then degraded at the metaphase of meiosis I and re-accumulates prior to meiosis II (S5A' Fig, [23,33]). As for Twine, CycB distribution was mostly unaffected by the loss of eIF4E-1 although we did observe some staining in the nuclei of the meiotic stages (S5B Fig and S5B' Fig). Taken together, these results show that eIF4E-1 is not required in primary spermatocytes for the progression of mitotic and meiotic cell divisions.

**Fig 3:**
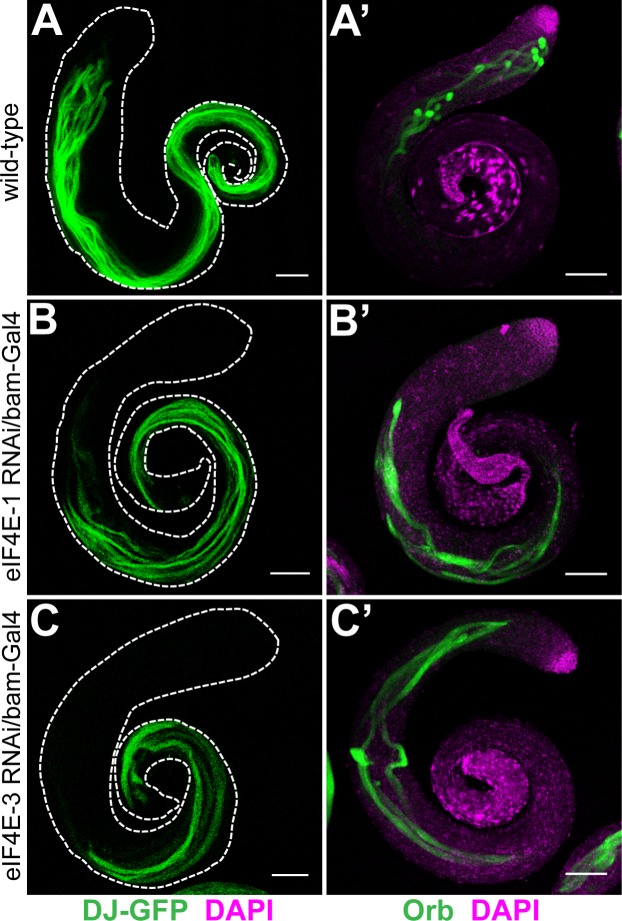
Distribution of Don Juan-GFP and Orb in *eIF4E-1* and *eIF4E-3* knockdown testes. GFP fluorescence of Don Juan-GFP (DJ-GFP) in the wild-type background (A) or testes expressing *eIF4E-1* (B) and *eIF4E-3* (C) shRNA under the *bam*-Gal4:VP16 driver. Whole mount anti-Orb staining (green) of wild-type (A') or *eIF4E-1* (B') and *eIF4E-3* (C') knockdown testes. All knockdowns were performed with shRNA driven by the *bam*-Gal4:VP16 driver. The testis outline is outlined with a dashed line in A-C. DAPI is shown in magenta. Scale bar 100 μm.

During maturation of the spermatid, condensation and shaping of the haploid nuclei occurs after completion of the cyst elongation phase. The nuclear condensation defect observed in the mature spermatids in testes expressing shRNA targeting *eIF4E-1* driven by *bam*-Gal4:VP16 could thus be due to aberrant spermiogenesis. We investigated this possibility by monitoring the localization of Orb and Don Juan (DJ) proteins. *orb* is transcribed post-meiotically and its mRNA localizes to the caudal tip of the elongating spermatid bundle [6,34]. However, it is translated only at the end of the elongation phase such that high levels of Orb are found at the caudal end of elongated spermatids undergoing nuclear condensation (Fig 3A', [35]). In testes expressing shRNA targeting *eIF4E-1* driven by *bam*-Gal4:VP16, Orb expression was not restricted to the tip of the elongated flagellar axoneme; instead the protein was distributed in a diffuse manner throughout the length of the elongated spermatid, which is indicative of a defective organization of the spermatid bundles (Fig 3B'). The mitochondria-associated protein DJ (visualized by DJ-GFP) is expressed later in the spermatid maturation phase in cysts undergoing individualisation; it is absent in spermatids that have not completed the elongation phase (Fig 3A, [36,37]). The distribution of DJ-GFP in *eIF4E-1* RNAi testes is relatively normal, indicating normal mitochondrial differentiation and that the spermatid cysts have completed elongation and initiated individualization. However, the elongated spermatids were consistently shorter than wild-type (compare Fig 3A with 3B).

**Fig 4:**
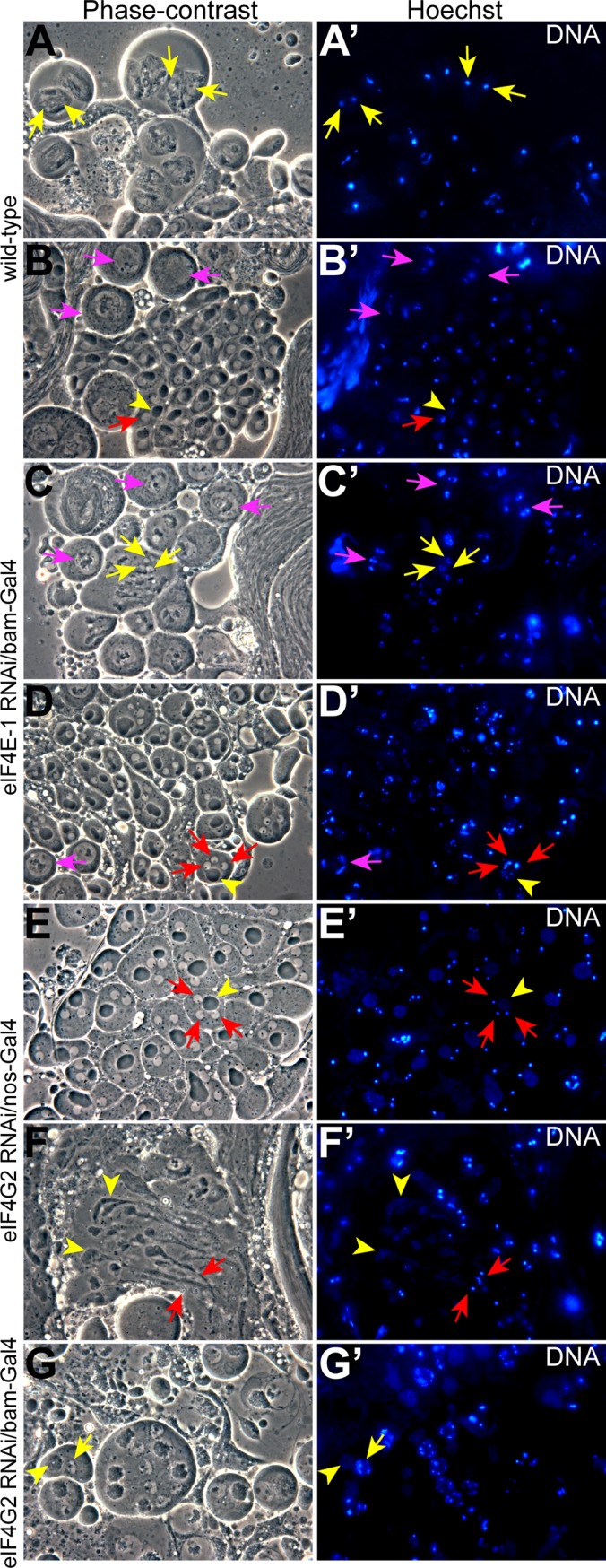
Effects of *eIF4E-1* and *eIF4G2* knockdown on spermatogenesis. Phase-contrast micrographs of squash preparations (left panel) and the corresponding Hoescht staining (right panel). As compared with the wild-type (A-B'), testes expressing shRNA targeting *eIF4E-1* (C-D') and *eIF4G2* (G) in the spermatocytes using *bam*-Gal4:VP16 driver show defects in nuclear condensation and cytokinesis, while knockdown of *eIF4G2* using *nos*-Gal4:VP16 driver (E-F') results in defective cytokinesis. The nuclei in prophase I, anaphase I and onion stage spermatids are marked by arrows in magenta, yellow and red, respectively. The arrowhead (yellow) shows the nebenkern in panels B & B', D & D' and E' & E' while the mitochondrial structure in elongated spermatids and spermatocytes is marked in F & F' and G & G', respectively. Note the three chromosome clumps in B', C' and D'.

Phase-contrast imaging of live squash preparations of testes expressing shRNA targeting *eIF4E-1* driven by *bam*-Gal4:VP16 revealed a defect in chromosome condensation and segregation during the meiotic stages. During the first meiotic division in the wild-type testes, the chromosomes in the spermatocyte nuclei organises into three clumps at the nuclear periphery (Fig 4B and 4B', magenta arrows), which rapidly coalesce at the onset of metaphase I to form a dot-like structure (stage M4, [38]) that persists during spermiogenesis until the onset of nuclear compaction (Fig 4A and 4A', yellow arrows,). In addition, each post-meiotic onion-stage haploid spermatid is characterized by a phase-dark mitochondrial aggregate, called the nebenkern (Fig 4B, arrowhead), that is closely associated with a phase-bright nucleus containing condensed chromatin (Fig 4B and 4B', red arrow). In testes expressing shRNA targeting *eIF4E-1* driven by *bam*-Gal4:VP16, M4 stage spermatocytes frequently had multiple nuclei, each containing dispersed chromatin structures (Fig 4C and 4C', yellow arrows). This fragmented chromatin organisation persisted through the onset of spermatid differentiation. Strikingly, a majority of the haploid onion-stage spermatids in these testes had more than one nucleus but only one nebenkern, and the flagellar axonemes failed to complete the process of individualisation (Fig 4D and 4D', red arrows). These data demonstrates an essential role for eIF4E-1 in regulating chromatin condensation and karyokinesis during meiotic divisions and post-meiotic differentiation processes. Notably, mitochondrial compaction and differentiation was not affected in these cysts (Fig 4D, arrowhead). Taken together, all these results indicate that several developmental functions in the later stages of spermiogenesis specifically require eIF4E-1.

Testes expressing *eIF4E-3* shRNA under *bam*-Gal4:VP16 control showed similar defects during spermatogenesis to those expressing *eIF4E-1* shRNA under *bam*-Gal4:VP16 control. As reported previously for the *eIF4E-3* mutant [17], *eIF4E-3* RNAi testes showed abnormal chromosome segregation and cytokinesis during meiosis (S6 Fig). Furthermore, we find that the distribution of eIF4E-1 and eIF4G2 proteins was unaffected in *eIF4E-3* knockdown testes (Fig 2D''' and S4C Fig and S4C' Fig). Twine and CycB proteins accumulated similar to wild-type, indicating successful G2/M transition of the primary spermatocytes into meiotic divisions (S5C–S5C'' Fig). However, post-meiotic development and spermatid differentiation were defective as revealed by the distribution of Orb and DJ proteins (Fig 3C and 3C'). The haploid nuclei failed to compact into needle-like bundles at the rostral end of the flagellar axoneme and were dispersed throughout the elongated spermatid cysts (Fig 2D'''' and S4C' Fig).

### eIF4G is required in the cyst cells for proper organisation of the germ cell cysts

To interrogate the function of eIF4G during spermatogenesis, we expressed shRNAs targeting its mRNA in the germ cells using the *nos*-Gal4:VP16 and *bam*-Gal4:VP16 drivers. Surprisingly, *eIF4G* knockdown males using either driver were fertile, and the testes did not show any detectable abnormality (S7 Fig). To test whether the expression of *eIF4G* shRNA effectively abrogates the corresponding transcript, we combined the *eIF4G* TRiP line with the *Ubi*-Gal4 driver, which is expressed globally in somatic tissues. We failed to obtain any viable adult progeny expressing the *eIF4G* shRNA. Furthermore, females expressing *eIF4G* RNAi with *nos*-Gal4:VP16 driver were sterile, with oogenesis arresting at pre-vitellogenic stages (S8 Fig). These results demonstrate that the shRNA produced from the *eIF4G* TRiP line results in a sufficient knockdown of the *eIF4G* transcript to produce phenotypes. Therefore, we conclude that eIF4G does not play an important role in germ cells.

**Fig 5:**
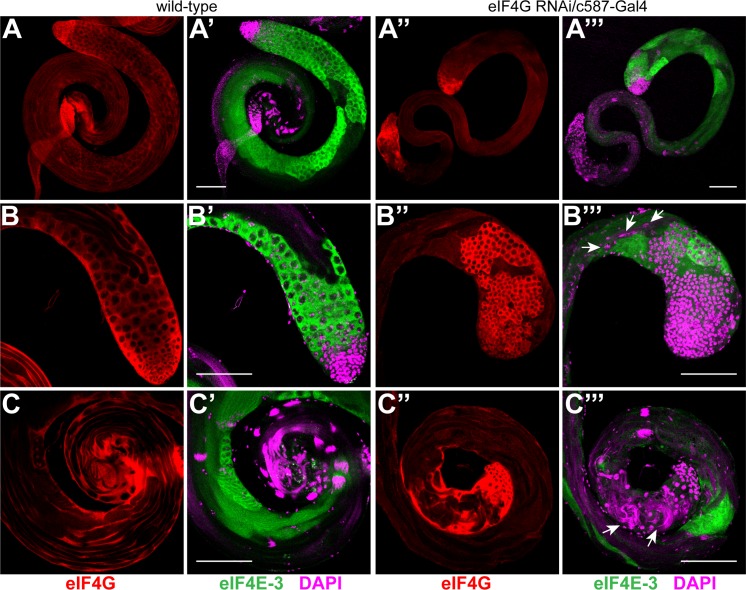
eIF4G is required in the somatic cyst cells for normal spermatogenesis. Wild-type testes (A-C') and testes expressing *eIF4G* shRNA with c587-Gal4 driver (A''-C''') were co-stained with anti-eIF4G (red) and anti-eIF4E-3 (green) antibodies. Loss of anti-eIF4G staining in the cyst cells at the apical tip of testis (B'') indicates efficient knockdown of the protein. The testis shows an increased number of abnormal germ cell cysts at its apical tip (B'') and reduced meiotic and post-meiotic cysts along its length (compare A' & C' with A''' & C''') as revealed by anti-eIF4E-3 staining (green). Although mature sperm nuclear bundles are found at the distal end of the testes, they appear disorganised (C''', arrows) and nuclei at various stages of condensation are also found at the caudal end of the elongating spermatids (B''', arrows).

In contrast, expression of *eIF4G* shRNA in somatic cyst cells using the c587-Gal4 driver affected male fertility. As compared with wild-type, c587-Gal4-*eIF4G* males produced significantly fewer progeny. Anti-eIF4G staining of RNAi testes showed efficient knockdown of the protein in the cyst cells at the apical tip (compare Fig 5B with 5B''). In a majority of the mutant testes we observed decreased spatial organization and over-proliferation of the primary spermatocyte cysts (Fig 5B''and 5B'''). As compared to the wild-type testes, meiotic and post-meiotic stages were under-represented (Fig 5A'''–5C''') and sperm maturation also appeared to be affected in *eIF4G* knockdown testes. Nuclei with varying degrees of condensation were found dispersed in the elongated spermatids, and needle-like sperm nuclear bundles were detected both at the caudal and rostral end of the sperm tails (Fig 5B''' and 5C''', arrows). This implicates somatic eIF4G as essential during spermatogenesis.

### eIF4G2 is essential for progression of the meiotic stages

Based on analysis of mutants, the testes-specific translation factor eIF4G2 has been shown to be required for spermatocytes to enter meiosis and differentiate into spermatids, and consequently for male fertility [18,19]. Since eIF4G2 is expressed both in the somatic cyst and in germ cells (Fig 1E'–1E''), we wanted to distinguish its function in the two cell lineages as well as probe its role during the different phases of germ cell development.

**Fig 6:**
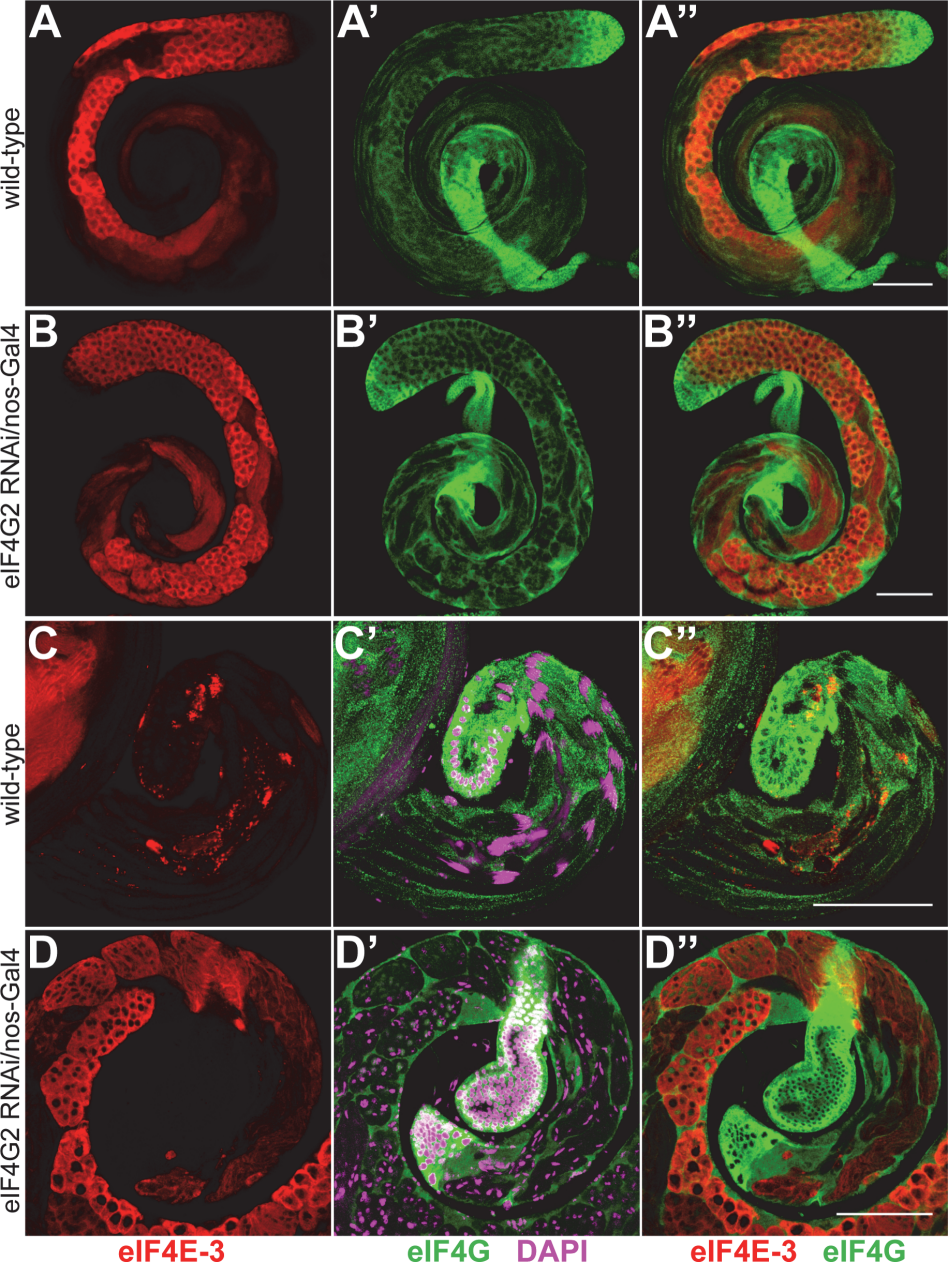
Knockdown of eIF4G2 in early stages affects meiotic divisions and differentiation during spermatogenesis. Wild-type testes (A-A'', C-C'') and testes expressing *eIF4G2* shRNA under *nos*-Gal4:VP16 control (B-B'', D-D'') were co-stained with anti-eIF4E-3 (red) and anti-eIF4G (green). Panels A-B'' show entire testes while the distal end of the testes is shown C-D''. The merged images are shown in A''-D''. DAPI staining is shown in magenta. Note the absence of elongated flagellar axonemes and nuclear bundles in the eIF4G2 knockdown testes (D') as compared with the wild-type (C'). Scale bar 100 μm.

Knockdown of *eIF4G2* using the cyst cell driver c587-Gal4 did not produce defects during spermatogenesis or affect fertility in the males indicating that somatic cyst expression of eIF4G2 is not essential (S9B–S9B'' Fig). In contrast, expression of *eIF4G2* shRNA under the control of *nos*- or *bam*-Gal4:VP16 driver resulted in male sterility. However, the knockdowns affected testes development differently than was observed for the *eIF4G2* mutant. Knockdown of *eIF4G2* using *nos*-Gal4:VP16 did not appear to affect the distribution of eIF4E-3 or spermatocyte cyst development (compare Fig 6A with 6B), but the elongated spermatids in the late stages of differentiation that encompass the length of the wild-type testes were absent (compare Fig 6A' and 6C' with 6B' and 6D'). In contrast to the *eIF4G2* mutant, we consistently observed meiotic and post-meiotic stage cysts, but the spermatid cysts appeared to be arrested in the early elongation phase and degenerated thereafter (Fig 6D'–6D''). Phase squash preparations of testes expressing shRNA targeting *eIF4G2* using *nos*-Gal4:VP16 showed multiple nuclei in haploid onion-stage spermatids, which appeared larger than the wild-type cysts (Fig 4E and 4E', red arrows), suggesting aberrant chromosome segregation and cytokinesis during the meiotic divisions. Notably, nuclear condensation during the mitotic and meiotic phases was not affected by the loss of eIF4G2. In addition, the mitochondria seem to unfurl and enter the differentiation program (Fig 4F and 4F'). We conclude that *eIF4G2* function in the early stages of spermatogenesis, when the *nos* driver is active, is necessary for meiotic cell divisions and completion of spermiogenesis.

**Fig 7:**
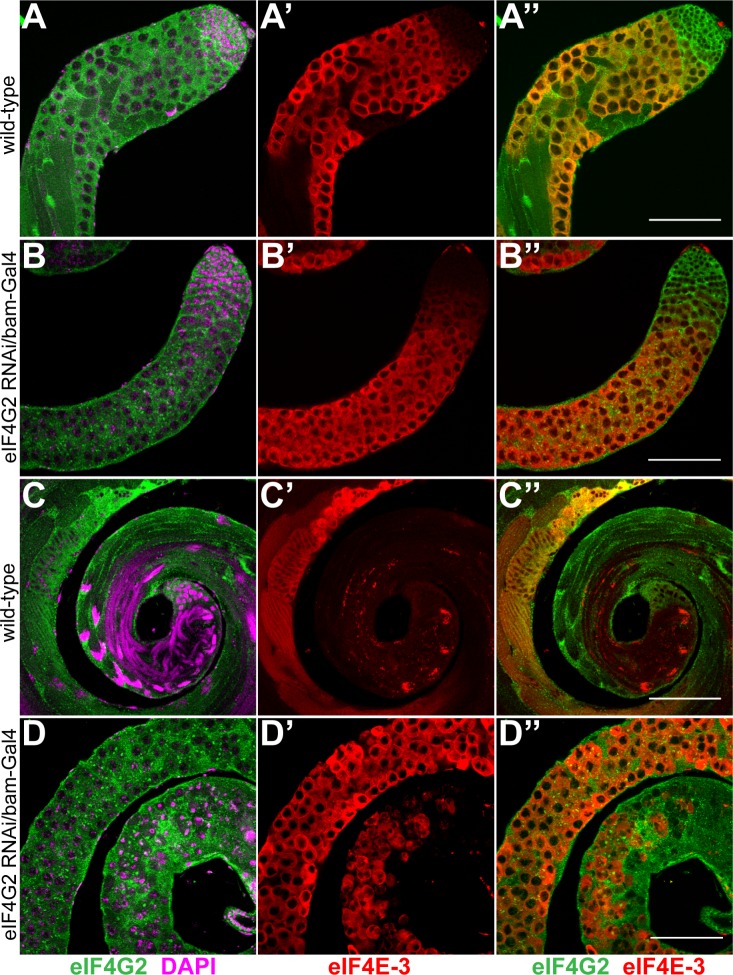
eIF4G2 function in the spermatocytes is essential for meiotic divisions during spermatogenesis. Co-staining of wild-type testes (A-A'', C-C'') and testes expressing shRNA targeting *eIF4G2* using *bam*-Gal4:VP16 driver (B-B'', D-D'') with anti-eIF4G2 (green) and anti-eIF4E-3 (red) antibody. Knockdown of *eIF4G2* in the spermatocytes does not affect eIF4E-3 distribution at the apical tip of the testes (compare A' with B') but causes meiotic arrest and results in degenerating germ cells at the distal end (D-D''). The merged images are shown in A''-D''. DNA is stained with DAPI (magenta). Scale bar 100 μm.

Unlike for *nos*-Gal4:VP16, when *bam*-Gal4:VP16 was used to knock down *eIF4G2* in primary spermatocytes, we failed to detect meiotic or post-meiotic elongated spermatid cysts, similar to observations in *eIF4G2* mutant testes. Instead, the testes were mostly filled with large mature spermatocytes which degenerated at the distal tip (compare Fig 7C' and 7C'' with 7D' and 7D''). Consistent with this, CycB was not detected in the cytoplasm of the cells (S5D' Fig) while Twine positive cells were found until the distal end of the testes (S5D'' Fig). Later spermatid stages with elongated flagella were absent in the RNAi testes. Phase contrast microscopy of live testes squashes revealed mature primary spermatocytes arrested at the G2/M transition. In contrast to wild-type, the nuclei contained dispersed chromatin and the mitochondria failed to fuse fully (Fig 4G and 4G'). Importantly, the nucleus to mitochondrion ratio was not affected, indicating normal cytokinesis. In addition we observed a few elongated cells in our preparations that appeared abnormal and did not stain with Hoescht. Therefore, eIF4G2 is required in spermatocytes for entry into meiosis and later phases of spermatid differentiation.

### eIF4G and eIF4G2 act redundantly in testes morphogenesis

**Fig 8:**
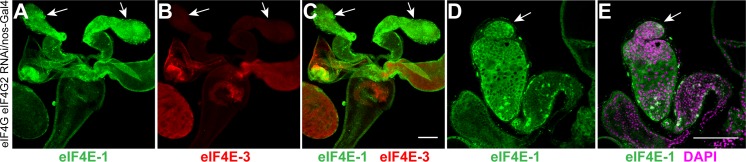
eIF4G and eIF4G2 act redundantly during early spermatogenesis. Knockdown of *eIF4G* and *eIF4G2* using the *nos*-Gal4:VP16 driver results in a rudimentary testes structure (marked by arrows). Anti-eIF4E-1 (A) and anti-eIF4E-3 (B) immunostainings are shown in green and red, respectively, while the merged image is shown in C. Absence of eIF4E-3 positive cells indicates spermatogenesis arrest at the spermatogonia stage. The magnified image of the apical tip of the testis (arrow, D, E) shows a mass of cells. DNA is stained with DAPI (magenta). Scale bar 100 μm.

As described above, expression of eIF4E-1 in the hub cells and spermatogonial stages is essential for the development of germ cells and consequently testes morphogenesis (Fig 2B' and 2C'). However, a lack of a similar requirement for either eIF4G or eIF4G2, proteins that bind eIF4E-1 and are essential for translation, was intriguing. To explore the possibility that eIF4G and eIF4G2 function redundantly during the early stages of spermatogenesis, we generated flies expressing shRNAs targeting both genes under the *nos* promoter. These males were sterile and produced rudimentary testes similar to testes expressing shRNA targeting *eIF4E-1* under the *nos* promoter (Fig 8A–8C). The apical tips of the testes were filled with masses of dividing cells, indicating an arrest before the primary spermatocyte stage (Fig 8D and 8E). This phenotype indicates functional redundancy of eIF4G and eIF4G2 during early spermatogenesis and argues against a functional role for other eIF4G-like proteins in male germ cell differentiation.

## Discussion

Post-transcriptional control of gene expression is important in regulating cellular processes during germline and embryonic development [13]. Although testes-specific translation initiation factors eIF4E-3 and eIF4G2 are required for male fertility [17–19], their roles during the different stages of spermatogenesis have not been addressed before, and the role of eIF4E-1 and eIF4G in testes development remained unknown. Using gene knockdown in specific domains of the testes, our analysis unveils diverse functions of these initiation factors in the germline and soma.

Variant isoforms of the cap-binding protein eIF4E in different organisms are involved in specialized functions. In mammals, an eIF4E isoform called eIF4E-3 does not bind 4E-BP and cannot functionally rescue a yeast mutant in eIF4E-1 (TIF1) although it binds eIF4G [39]. In zebrafish, two eIF4E isoforms are differentially expressed, one is ubiquitous while the other is specific to gonads and early embryos [40]. A germline-specific function has been shown for *C*. *elegans* IFE-1 and -2 [41,42]. The *Drosophila* genome encodes seven eIF4E genes which encode proteins similar to eIF4E-1 [16] [43]. Four of these the eIF4E paralogs (eIF4E-3, -4, -5, and -7) are almost exclusively expressed in the testes [44]. Indeed, eIF4E-3 is required in the primary spermatocytes for chromosome condensation and segregation during meiosis and later stages of spermiogenesis (this study, [17]). Perhaps surprisingly, in this study we found that lack of eIF4E-1 in the spermatocytes causes similar defects to loss of eIF4E-3. Since knockdown of either eIF4E-1 or eIF4E-3 did not detectably affect the amounts of the other isoform (S4D Fig), this suggests that these two cap-binding proteins are essential for translation of different transcripts in the spermatocytes, perhaps including mRNAs encoding cell cycle regulators necessary to ensure proper G2/M transition. As the cap structure is not known to be heterogeneous among testes-expressed mRNAs, eIF4E-1 and eIF4E-3 could potentially associate preferentially with different sets of mRNAs through association with different partner proteins that directly or indirectly bind mRNA with some specificity. The observation that eIF4E-1, but not eIF4E-3, can interact with 4E-BP is consistent with this hypothesis [17]. Regardless of the underlying mechanism, our data indicate that both eIF4E-1 and eIF4E-3 are required for spermatid elongation and nuclear shaping events, crucial for formation of the mature sperm.

In addition, our results demonstrate that during early spermatogenesis, eIF4E-1 is required both in the germline and soma for the normal development of testes. We postulate that absence of eIF4E-1 is lethal for somatic cells, and the absence of somatic cells leads to arrest of germ cell development. Such a requirement of factors in the somatic cyst cells regulating gonadogenesis and germ cell identity has been reported [29,45]. Interestingly, somatic expression of eIF4G during similar stages is required for germ cell organization and polarity of the elongating spermatid cysts.

While germline-specific knockdown of eIF4G gave no phenotype, we observed a much more severe phenotype when both eIF4G and eIF4G2 were knocked down in germ cells than when eIF4G2 alone was knocked down. This suggests that eIF4G can functionally substitute for eIF4G2 in pre-meiotic stages of spermatogenesis. *Drosophila* eIF4G and eIF4G2 share substantial amino acid sequence identity in the conserved middle domain and bind PABP (S10 Fig), however, eIF4G2 has a poorly conserved N-terminal domain and lacks a typical eIF5-CTD domain, making its ability to functionally substitute for eIF4G somewhat surprising. Our analysis nevertheless establishes eIF4G2 as the principal factor for translation initiation in spermatocytes and post-meiotic spermatids and points towards the formation of a specialized eIF4F complex that is necessary for translation of testes-specific transcripts during development. This is consistent with results from human cells in which an isoform-specific role of eIF4GII has been observed during the G2/M phase of mitosis [46].

Our results, taken together with biochemical data reported previously [17] support the existence of specialized eIF4F complexes in spermatocytes and post-meiotic spermatids. At the onset of spermatogenesis, we propose that eIF4E-1 interacts with either eIF4G or eIF4G2 to translate mRNAs required for progression to the spermatocyte stage. During spermatocyte maturation, translation mediated by the eIF4E-1-eIF4G2 and eIF4E-3-eIF4G2 complexes is essential for proper chromosome condensation and G2/M transition necessary for entry into meiosis. Upon completion of meiosis, these eIF4F complexes are required either for translation of the repressed messages or newly synthesized mRNAs that dictate spermatid maturation resulting in the formation of functional gametes.

Genome-wide analysis has revealed sex-biased gene expression in *Drosophila*, with male germ cells expressing significantly more transcripts than female germ cells [44,47]. Furthermore, gene expression during different stages of spermatogenesis has been profiled [48]. However, the translation status and roles of the testes-specific eIF4F complexes remain to be addressed. In the future, using RNA-immunoprecipitation-RNA sequencing, it would be possible to determine the direct mRNA targets of the different eIF4F complexes. Together with proteomic analysis of the knockdown testes, this should provide new insights into mechanisms of translation regulation and how they impact germline development.

## Supporting Information

S1 Fig eIF4E-1 and eIF4G2 are expressed in the soma and germline while eIF4E-3 is germ cell-specific. (A-A'') The overlay of anti-eIF4E-1 and anti-eIF4E-3 staining as shown in Fig 1. Fluorescent *in situ* hybridisation with the sense (B, B') and antisense (C, C') *eIF4E-3* probe shows absence of the mRNA from the apical tip of testes and enrichment of it in spermatocytes. Scale bar 100 μm. Magnified image of the mature spermatocyte cysts shows presence of eIF4E-1 (D, green) and eIF4G2 (E, green) proteins in the cytoplasm of the spermatocytes and the surrounding cyst cells (arrow) while eIF4E-3 (D', E', red) is restricted to the germ cell cytoplasm. The merged images are shown in D'' and E''. Scale bar 20 μm. Co-staining of the wild-type testes (F, G) and testes expressing Sa-GFP (F', G') with anti-GFP and anti-eIF4E-3 antibodies show considerable overlap of expression of eIF4E-3 with Sa-GFP, a marker of primary spermatocytes, at the apical tip of testes. Scale bar 100 μm.
(TIF)

S2 Fig Expression domains of the *nos*-Gal4:VP16 and *bam*-Gal4:VP16 in testes. Distribution of GFP fluorescence in testes using *nos*-Gal4:VP16 (A, B) and *bam*-Gal4:VP16 (C, D) drivers expressing UAS-GFP show that the *nos* promoter activity is restricted to the apical tip of the testes in the region containing the hub cells and spermatogonia, while the *bam* promoter is active in the spermatocyte cysts. Note the absence of GFP signal from the apical tip of the testes in D. The testis outline is outlined with a dashed line. Scale bar 100 μm.
(TIF)

S3 Fig Knockdown of *eIF4E-1* in spermatocytes does not affect the distribution of eIF4E-3. Co-staining of wild-type testes (A-A'' & C-C'') and testes expressing *eIF4E-1* RNAi under the *bam*-Gal4:VP16 driver (B-B'' & D-D'') with anti-eIF4E-1 (green) and anti-eIF4E-3 (red) antibody reveals specific knockdown of eIF4E-1 in the spermatocytes and a normal distribution pattern of eIF4E-3. The right panel shows the merged images of anti-eIF4E-1 and anti-eIF4E-3 staining (A''-D''). The apical end of the testes containing the spermatocytes is depicted in A-B'' while the post-meiotic stages are shown in C-D''. Scale bar 100 μm.
(TIF)

S4 Fig Knockdown of *eIF4E-1* and *eIF4E-3* in spermatocytes does not affect the distribution of eIF4G2. Anti-eIF4G2 staining (green) of the wild-type testes (A, A') and testes expressing *eIF4E-1* RNAi (B, B') and *eIF4E-3* RNAi (C, C') under the *bam*-Gal4:VP16 driver shows normal distribution pattern of eIF4G2 protein. Note the absence of needle-like nuclear bundles (marked with arrows in wild-type, A') at the distal tip of the testes expressing *eIF4E-1* (B') and *eIF4E-3* (C') RNAi. DAPI is shown in magenta. Scale bar 100 μm. (D) Western blot analysis of extracts from testes expressing *eIF4E-1* and *eIF4E-3* RNAi in the spermatocytes shows efficient knockdown of the corresponding proteins. Furthermore, the levels of eIF4E-1 and eIF4E-3 in the *eIF4E-3* and *eIF4E-1* knockdown testes, respectively, remains largely unaffected.
(TIF)

S5 Fig Distribution of CyclinB-GFP and Twine-LacZ in testes expressing *eIF4E-1*, *eIF4E-3* and *eIF4G2* shRNA in spermatocytes. Confocal scanning micrographs showing GFP fluorescence (CycB-GFP) in wild-type (A, A') or *eIF4E-1* (B, B'), *eIF4E-3* (C, C') and *eIF4G2* (D, D') knockdown testes. The apical end is shown in A-D while the mature spermatocyte and meiotic stages are shown in A'-D'. Scale bar 100 μm. Bright field micrographs showing Twine-LacZ distribution (shown in dark blue) in the wild-type background (A'') or *eIF4E-1* (B''), *eIF4E-3* (C'') and *eIF4G2* (D'') knockdown testes as revealed by the β-galactosidase activity assay. All knockdowns were performed with shRNA driven by the *bam*-Gal4:VP16 driver.
(TIF)

S6 Fig eIF4E-3 is essential for chromosome condensation and cytokinesis during the meiotic stages. Phase contrast microscopy (A, B) and the corresponding Hoescht staining (A', B') of testes expressing *eIF4E-3* RNAi using *bam*-Gal4:VP16 driver shows defective nuclear condensation and cytokinesis during the meiotic divisions that persists in the onion stage spermatids and later stages. Note the presence of multiple nuclei in the onion-stage spermatids (A & A', red arrows). The nebenkern and the unfurling mitochondria in post-meiotic cells are marked by arrowheads in A and B, respectively.
(TIF)

S7 Fig eIF4G function in the spermatocytes is dispensable for normal spermatogenesis. Wild-type testes (A-C') or testes expressing shRNA targeting *eIF4G* under *bam*-Gal4:VP16 control (A''-C'''') stained with anti-eIF4G antibody (green) shows specific knockdown of the protein in the spermatocytes while the surrounding cyst cells are unaffected (compare B with B'''). The top panels shows the entire testes (A-A'''') while the apical (B-B'''') and the distal (C-C'''') end of the testes are shown in the middle and lower panels, respectively. DAPI staining is shown in magenta. Scale 100 μm.
(TIF)

S8 Fig eIF4G is essential for oogenesis. (A) Knockdown of *eIF4G* using *nos*-Gal4:VP16 driver arrests oogenesis at the pre-vitellogenic stages resulting in female sterility. DAPI is shown in cyan.
(TIF)

S9 Fig Spermatogenesis is not affected by knockdown of *eIF4E-3* and *eIF4G2* in the somatic cyst cells. c587-Gal4 driven *eIF4E-3* (A-A'') and *eIF4G2* (B-B'') shRNA in the testes does not affect testes morphology or distribution of germ cells as revealed by staining with anti-eIF4E-1 (green) and anti-eIF4E-3 (red) antibody. The merged image is shown in A'' and B''. DNA is stained with DAPI (cyan). Note the presence of nuclear bundles at the distal end of the testes in A and B. Scale bar 100 μm.
(TIF)

S10 Fig eIF4G2 interacts with PABP *in vivo*. Wild-type testes extract was immunoprecipitated using IgG, anti-eIF4G and anti-eIF4G2 antibodies. The bound proteins (lanes 2, 3, 4) were western blotted and stained with the antibodies indicated at the right of the panel. The band corresponding to eIF4G2 is marked with an asterisk. Input (5%) is shown in lane 1.
(TIF)
