# Supplementary figures and images for: Loss-of-Function Analysis Reveals Distinct Requirements of the Translation Initiation Factors eIF4E, eIF4E-3, eIF4G and eIF4G2 in Drosophila Spermatogenesis
Source: PLoS One. 2015 Apr 7;10(4):e0122519. doi: 10.1371/journal.pone.0122519 (PMC4388691; doi:10.1371/journal.pone.0122519)

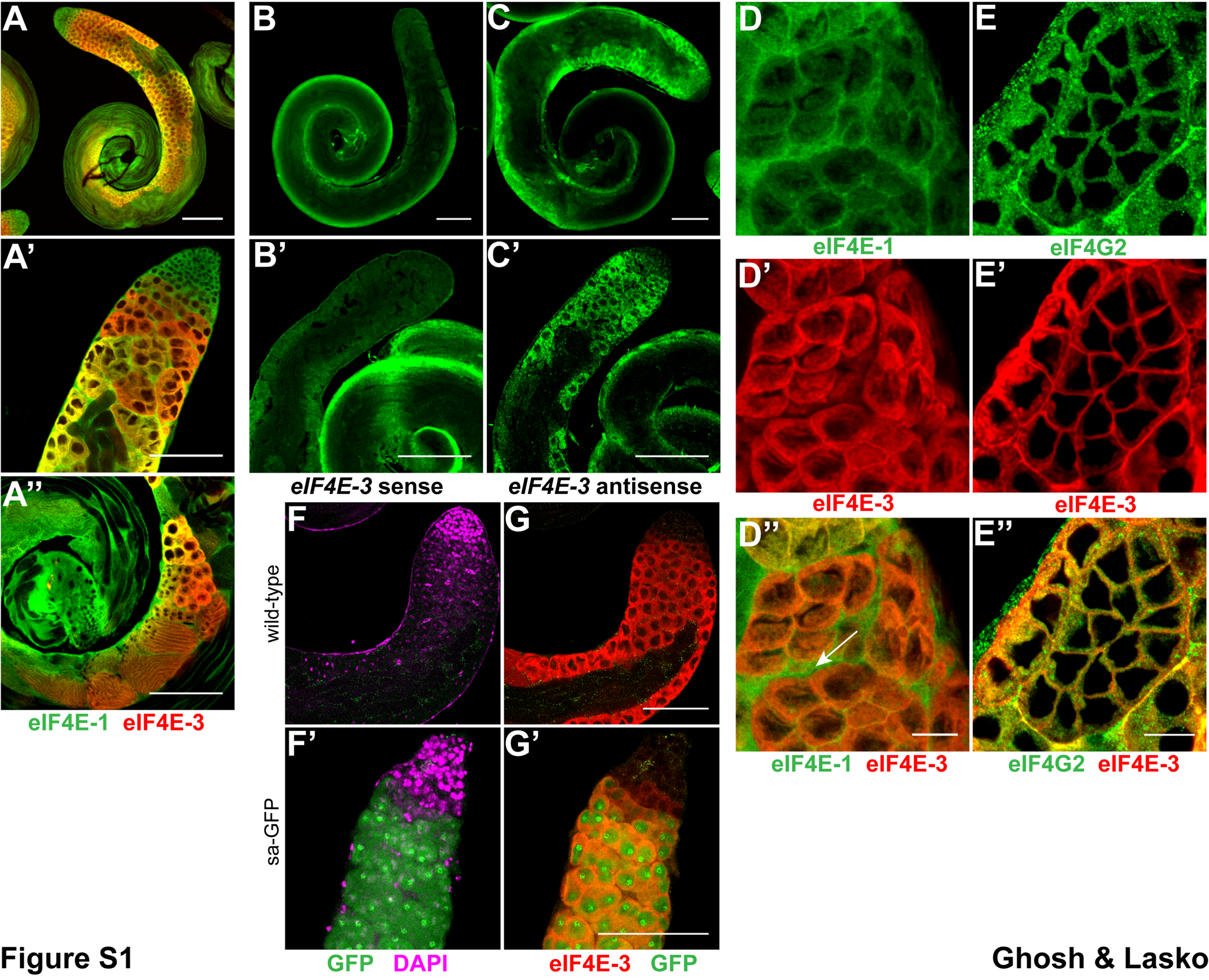

Supplement: S1 Fig — (A-A'') The overlay of anti-eIF4E-1 and anti-eIF4E-3 staining as shown in Fig 1. Fluorescent in situ hybridisation with the sense (B, B') and antisense (C, C') eIF4E-3 probe shows absence of the mRNA from the apical tip of testes and enrichment of it in spermatocytes. Scale bar 100 μm. Magnified image of the mature spermatocyte cysts shows presence of eIF4E-1 (D, green) and eIF4G2 (E, green) proteins in the cytoplasm of the spermatocytes and the surrounding cyst cells (arrow) while eIF4E-3 (D', E', red) is restricted to the germ cell cytoplasm. The merged images are shown in D'' and E''. Scale bar 20 μm. Co-staining of the wild-type testes (F, G) and testes expressing Sa-GFP (F', G') with anti-GFP and anti-eIF4E-3 antibodies show considerable overlap of expression of eIF4E-3 with Sa-GFP, a marker of primary spermatocytes, at the apical tip of testes. Scale bar 100 μm. (TIF) [file pone.0122519.s001.tif]

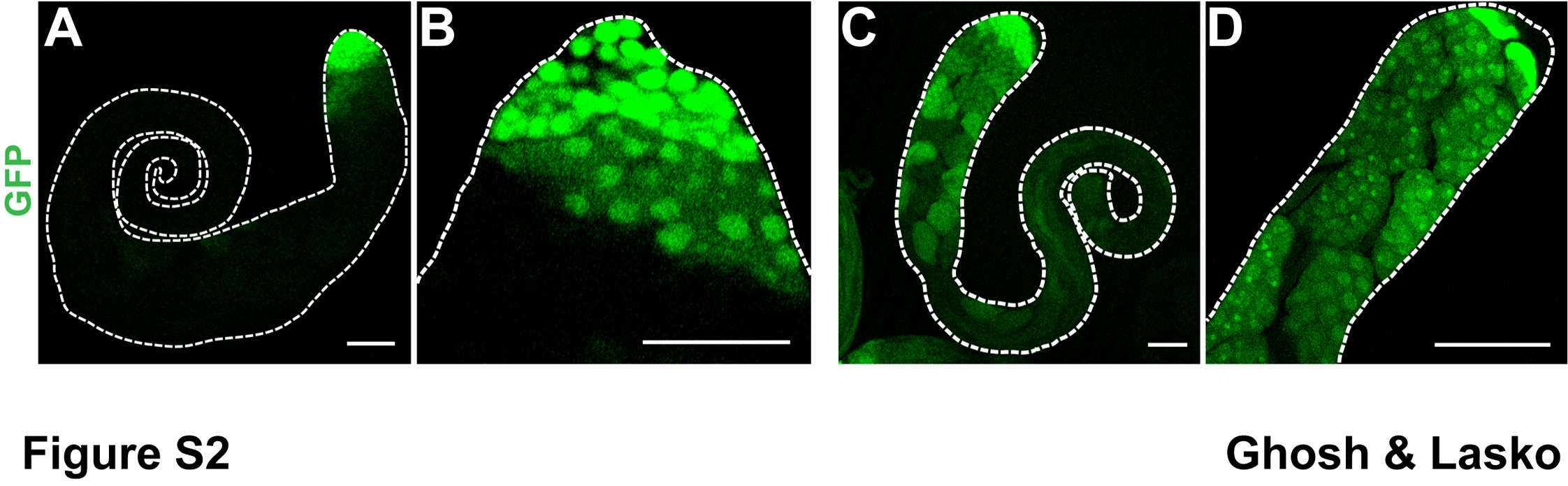

Supplement: S2 Fig — Distribution of GFP fluorescence in testes using nos-Gal4:VP16 (A, B) and bam-Gal4:VP16 (C, D) drivers expressing UAS-GFP show that the nos promoter activity is restricted to the apical tip of the testes in the region containing the hub cells and spermatogonia, while the bam promoter is active in the spermatocyte cysts. Note the absence of GFP signal from the apical tip of the testes in D. The testis outline is outlined with a dashed line. Scale bar 100 μm. (TIF) [file pone.0122519.s002.tif]

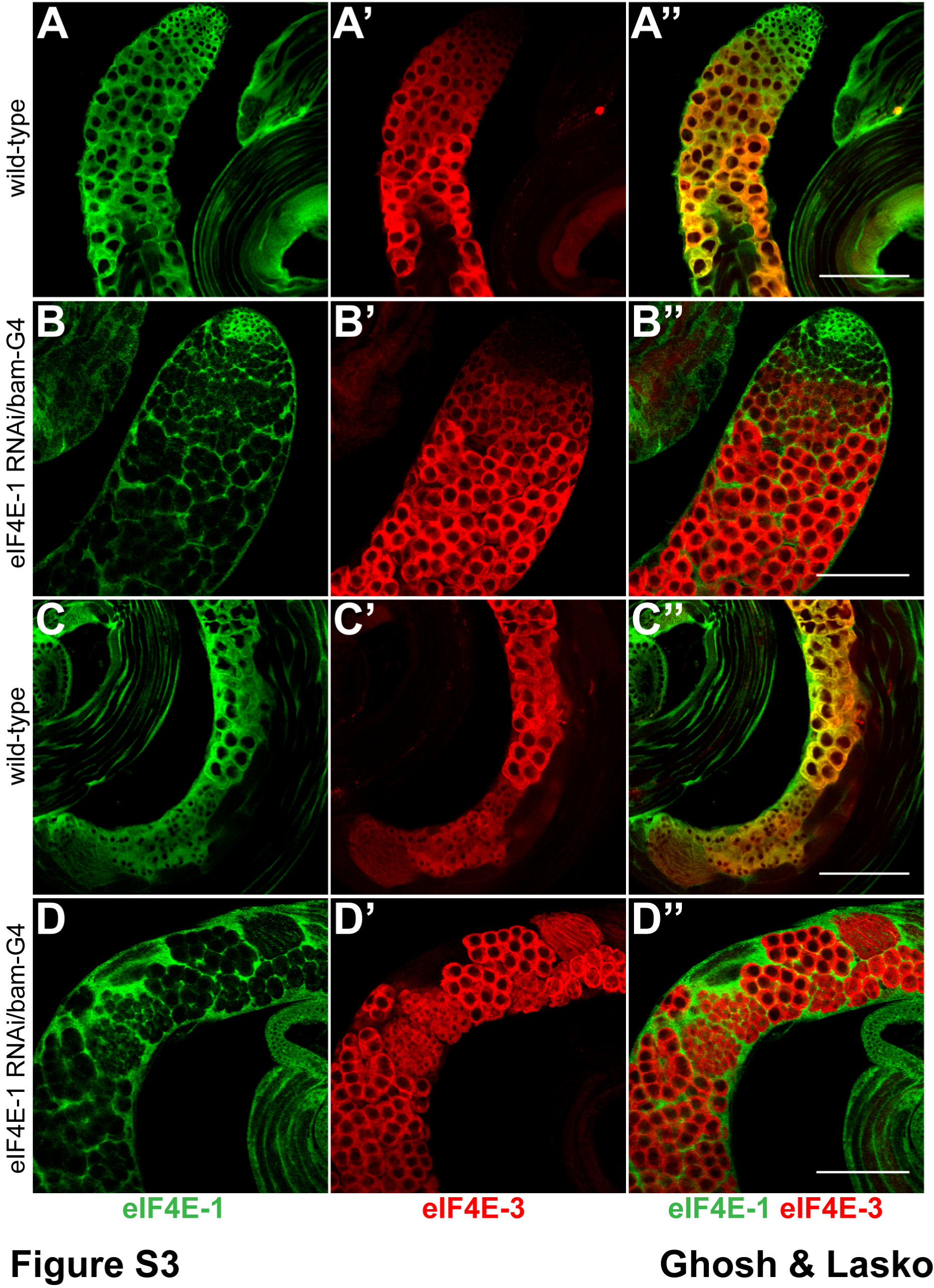

Supplement: S3 Fig — Co-staining of wild-type testes (A-A'' & C-C'') and testes expressing eIF4E-1 RNAi under the bam-Gal4:VP16 driver (B-B'' & D-D'') with anti-eIF4E-1 (green) and anti-eIF4E-3 (red) antibody reveals specific knockdown of eIF4E-1 in the spermatocytes and a normal distribution pattern of eIF4E-3. The right panel shows the merged images of anti-eIF4E-1 and anti-eIF4E-3 staining (A''-D''). The apical end of the testes containing the spermatocytes is depicted in A-B'' while the post-meiotic stages are shown in C-D''. Scale bar 100 μm. (TIF) [file pone.0122519.s003.tif]

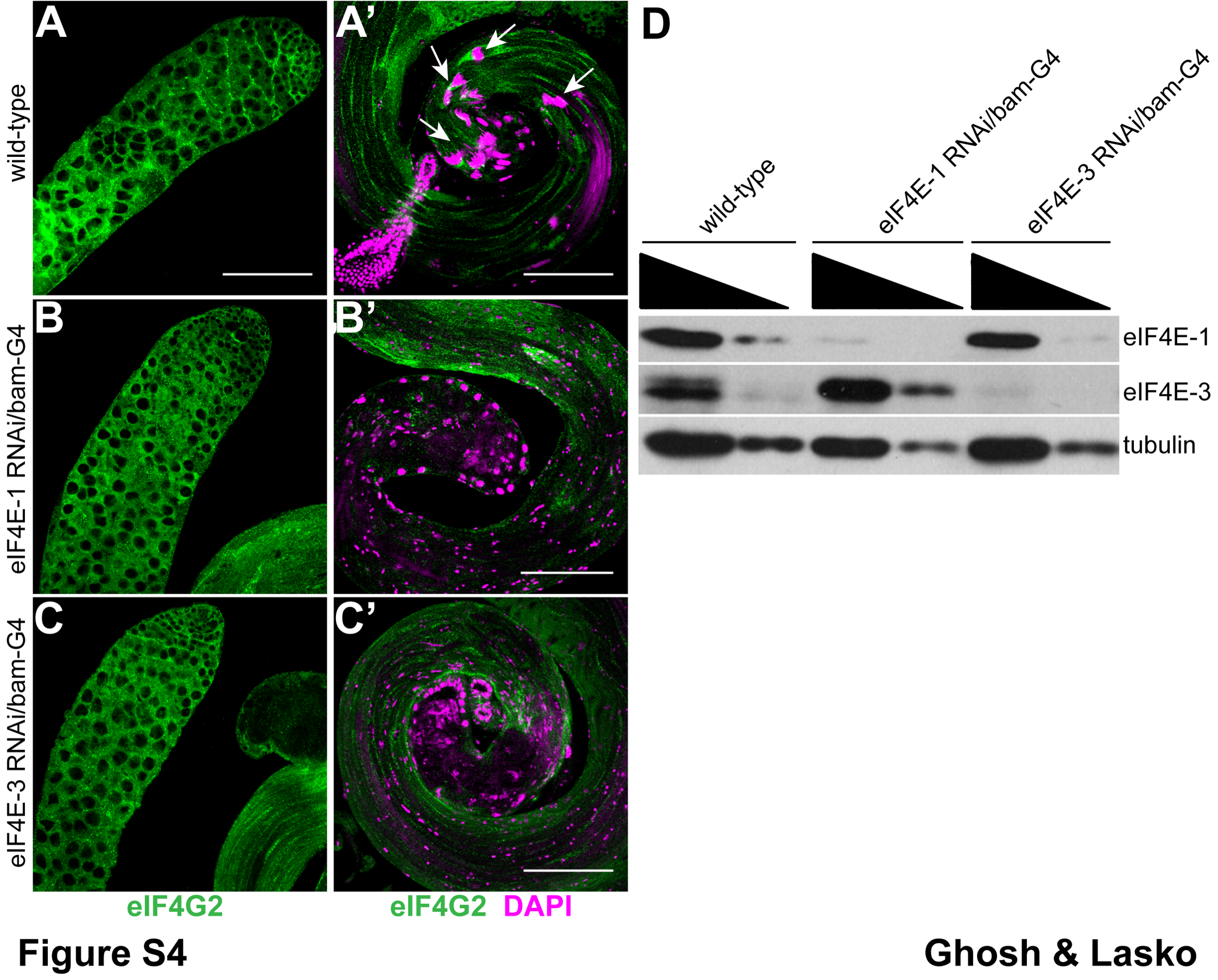

Supplement: S4 Fig — Anti-eIF4G2 staining (green) of the wild-type testes (A, A') and testes expressing eIF4E-1 RNAi (B, B') and eIF4E-3 RNAi (C, C') under the bam-Gal4:VP16 driver shows normal distribution pattern of eIF4G2 protein. Note the absence of needle-like nuclear bundles (marked with arrows in wild-type, A') at the distal tip of the testes expressing eIF4E-1 (B') and eIF4E-3 (C') RNAi. DAPI is shown in magenta. Scale bar 100 μm. (D) Western blot analysis of extracts from testes expressing eIF4E-1 and eIF4E-3 RNAi in the spermatocytes shows efficient knockdown of the corresponding proteins. Furthermore, the levels of eIF4E-1 and eIF4E-3 in the eIF4E-3 and eIF4E-1 knockdown testes, respectively, remains largely unaffected. (TIF) [file pone.0122519.s004.tif]

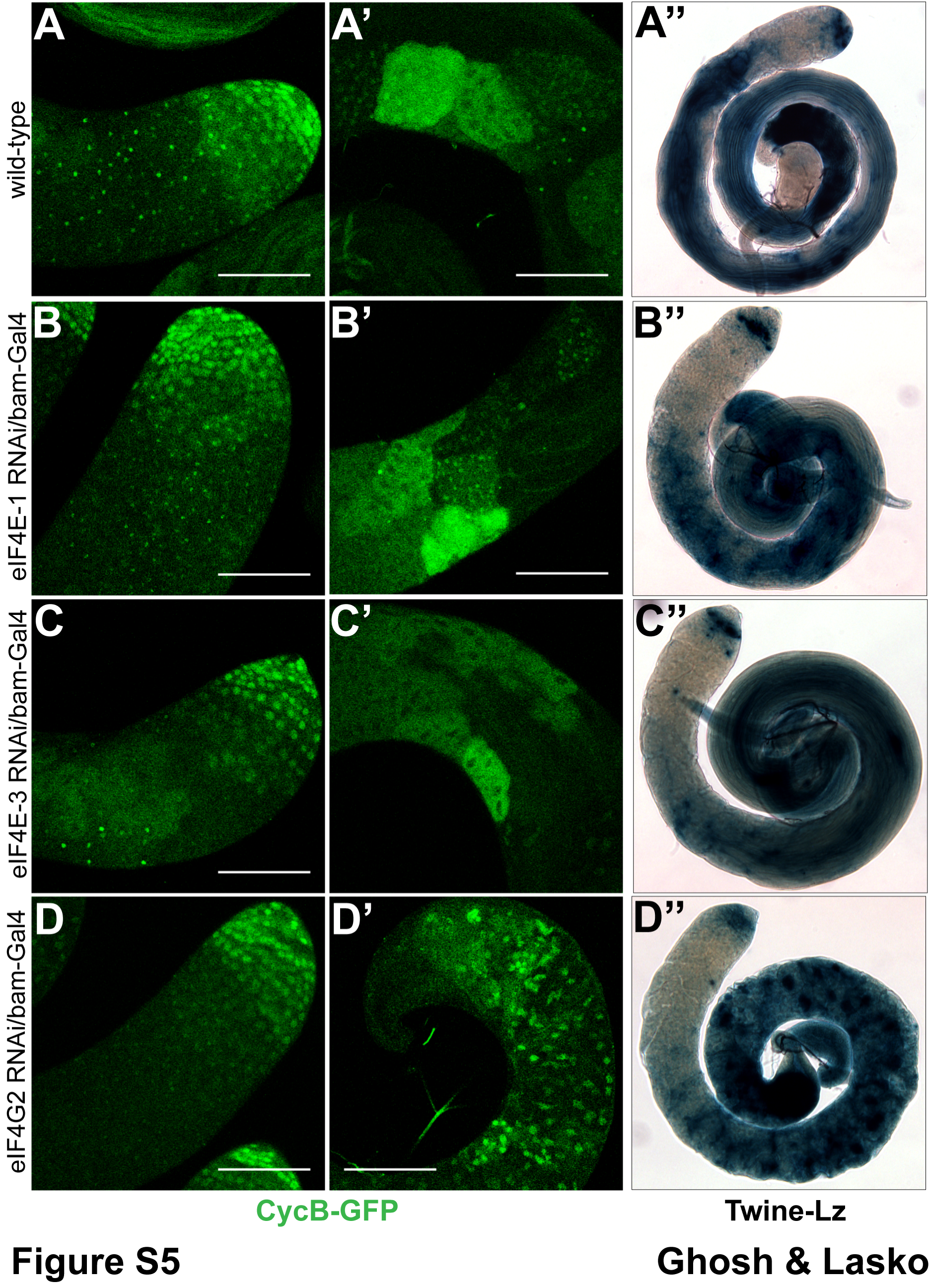

Supplement: S5 Fig — Confocal scanning micrographs showing GFP fluorescence (CycB-GFP) in wild-type (A, A') or eIF4E-1 (B, B'), eIF4E-3 (C, C') and eIF4G2 (D, D') knockdown testes. The apical end is shown in A-D while the mature spermatocyte and meiotic stages are shown in A'-D'. Scale bar 100 μm. Bright field micrographs showing Twine-LacZ distribution (shown in dark blue) in the wild-type background (A'') or eIF4E-1 (B''), eIF4E-3 (C'') and eIF4G2 (D'') knockdown testes as revealed by the β-galactosidase activity assay. All knockdowns were performed with shRNA driven by the bam-Gal4:VP16 driver. (TIF) [file pone.0122519.s005.tif]

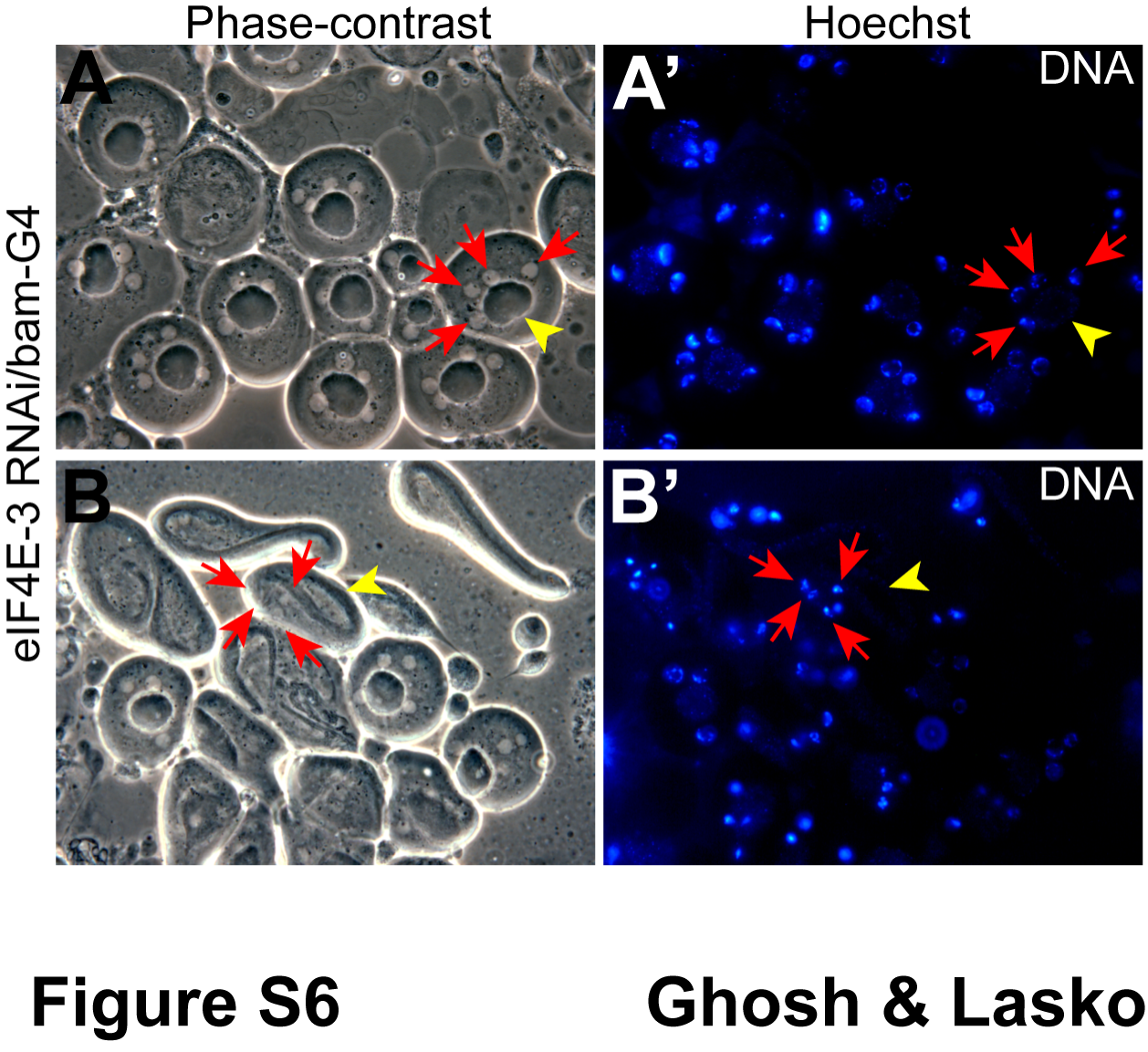

Supplement: S6 Fig — Phase contrast microscopy (A, B) and the corresponding Hoescht staining (A', B') of testes expressing eIF4E-3 RNAi using bam-Gal4:VP16 driver shows defective nuclear condensation and cytokinesis during the meiotic divisions that persists in the onion stage spermatids and later stages. Note the presence of multiple nuclei in the onion-stage spermatids (A & A', red arrows). The nebenkern and the unfurling mitochondria in post-meiotic cells are marked by arrowheads in A and B, respectively. (TIF) [file pone.0122519.s006.tif]

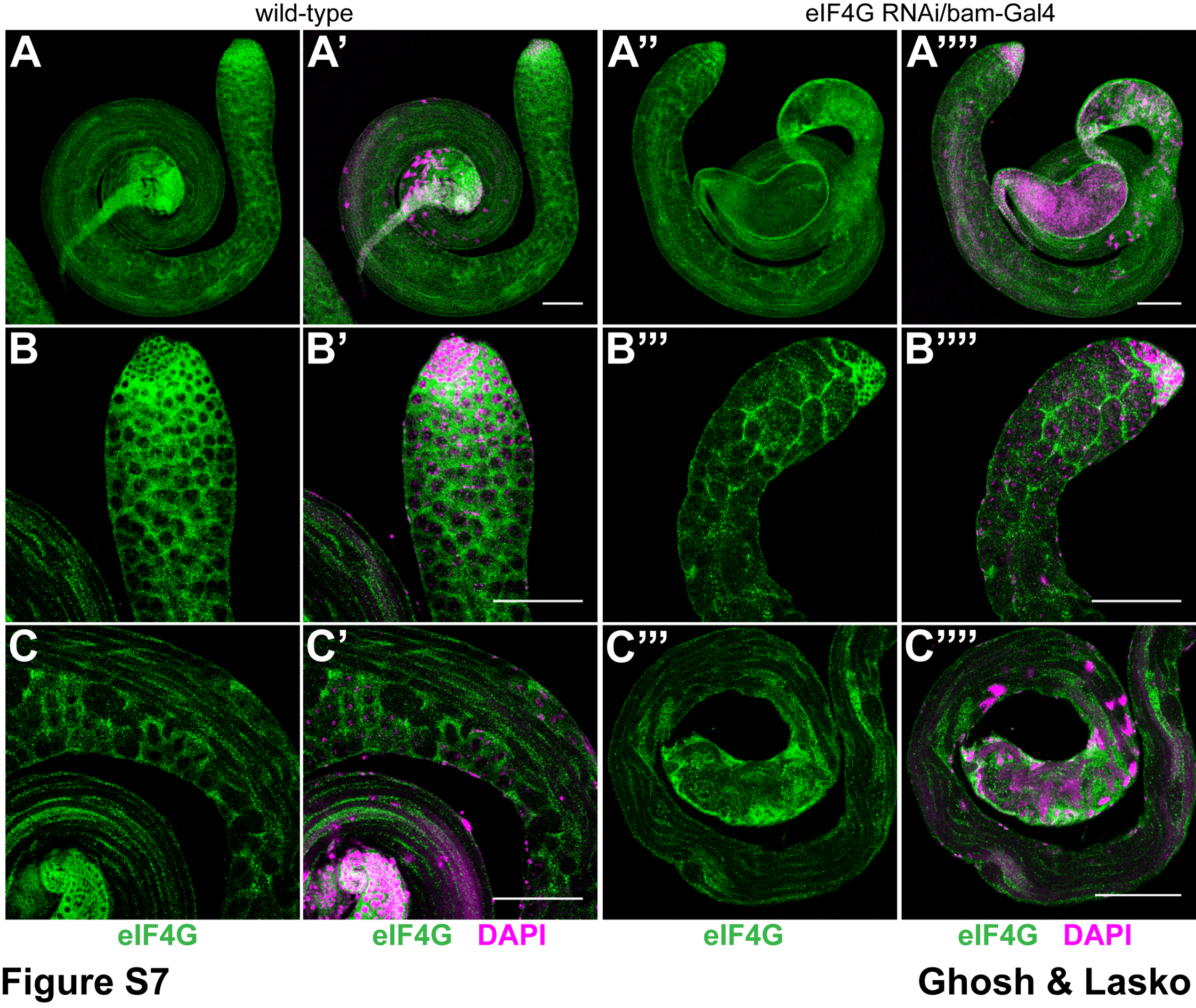

Supplement: S7 Fig — Wild-type testes (A-C') or testes expressing shRNA targeting eIF4G under bam-Gal4:VP16 control (A''-C'''') stained with anti-eIF4G antibody (green) shows specific knockdown of the protein in the spermatocytes while the surrounding cyst cells are unaffected (compare B with B'''). The top panels shows the entire testes (A-A'''') while the apical (B-B'''') and the distal (C-C'''') end of the testes are shown in the middle and lower panels, respectively. DAPI staining is shown in magenta. Scale 100 μm. (TIF) [file pone.0122519.s007.tif]

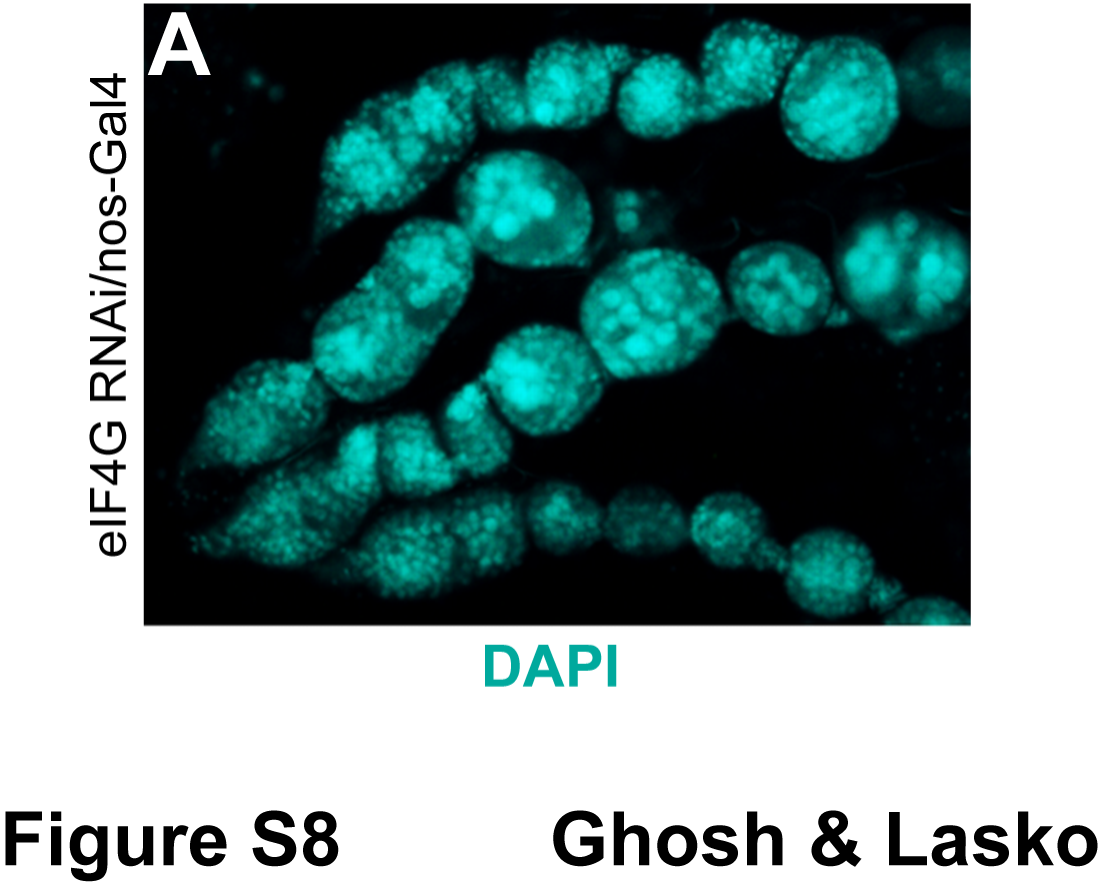

Supplement: S8 Fig — (A) Knockdown of eIF4G using nos-Gal4:VP16 driver arrests oogenesis at the pre-vitellogenic stages resulting in female sterility. DAPI is shown in cyan. (TIF) [file pone.0122519.s008.tif]

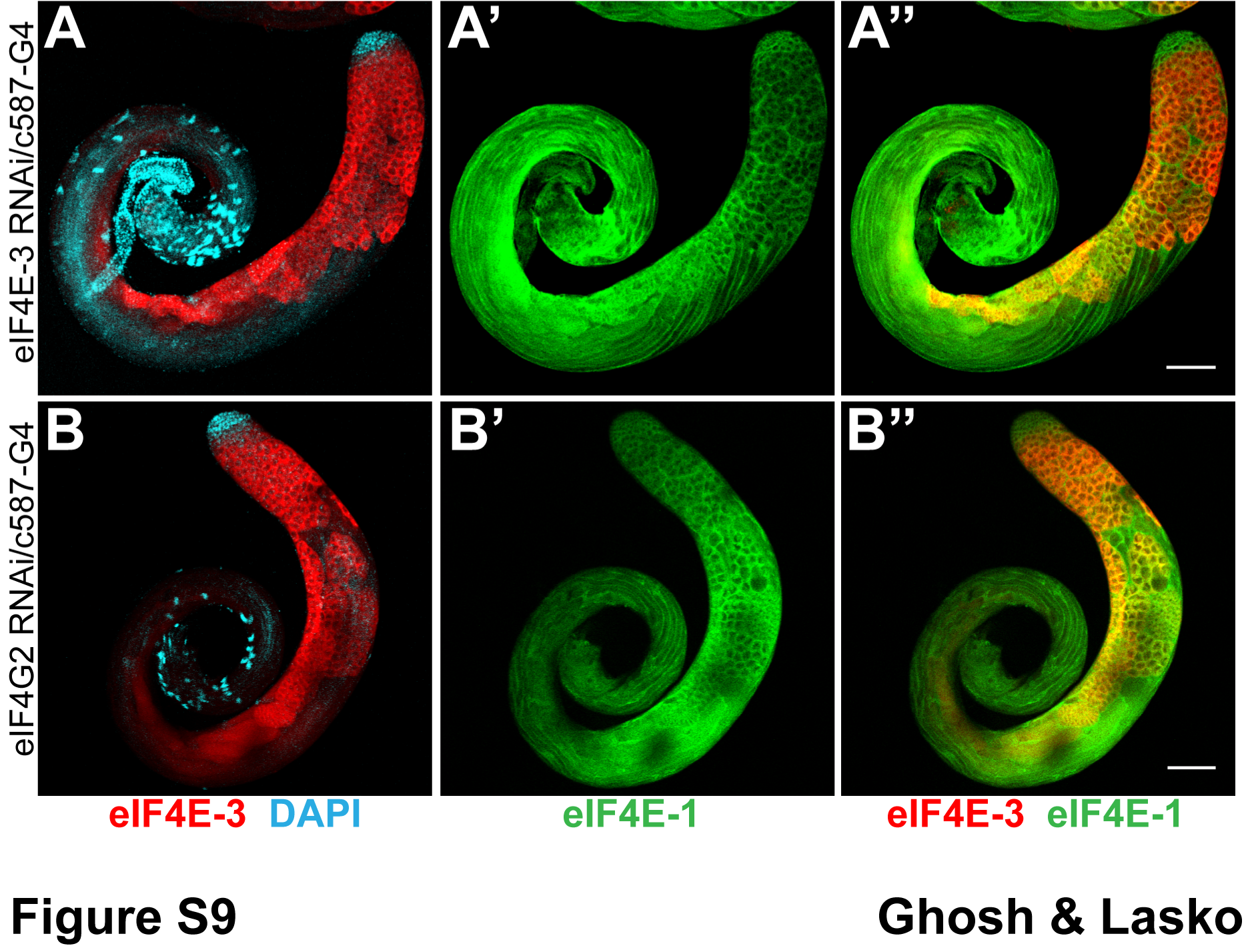

Supplement: S9 Fig — c587-Gal4 driven eIF4E-3 (A-A'') and eIF4G2 (B-B'') shRNA in the testes does not affect testes morphology or distribution of germ cells as revealed by staining with anti-eIF4E-1 (green) and anti-eIF4E-3 (red) antibody. The merged image is shown in A'' and B''. DNA is stained with DAPI (cyan). Note the presence of nuclear bundles at the distal end of the testes in A and B. Scale bar 100 μm. (TIF) [file pone.0122519.s009.tif]

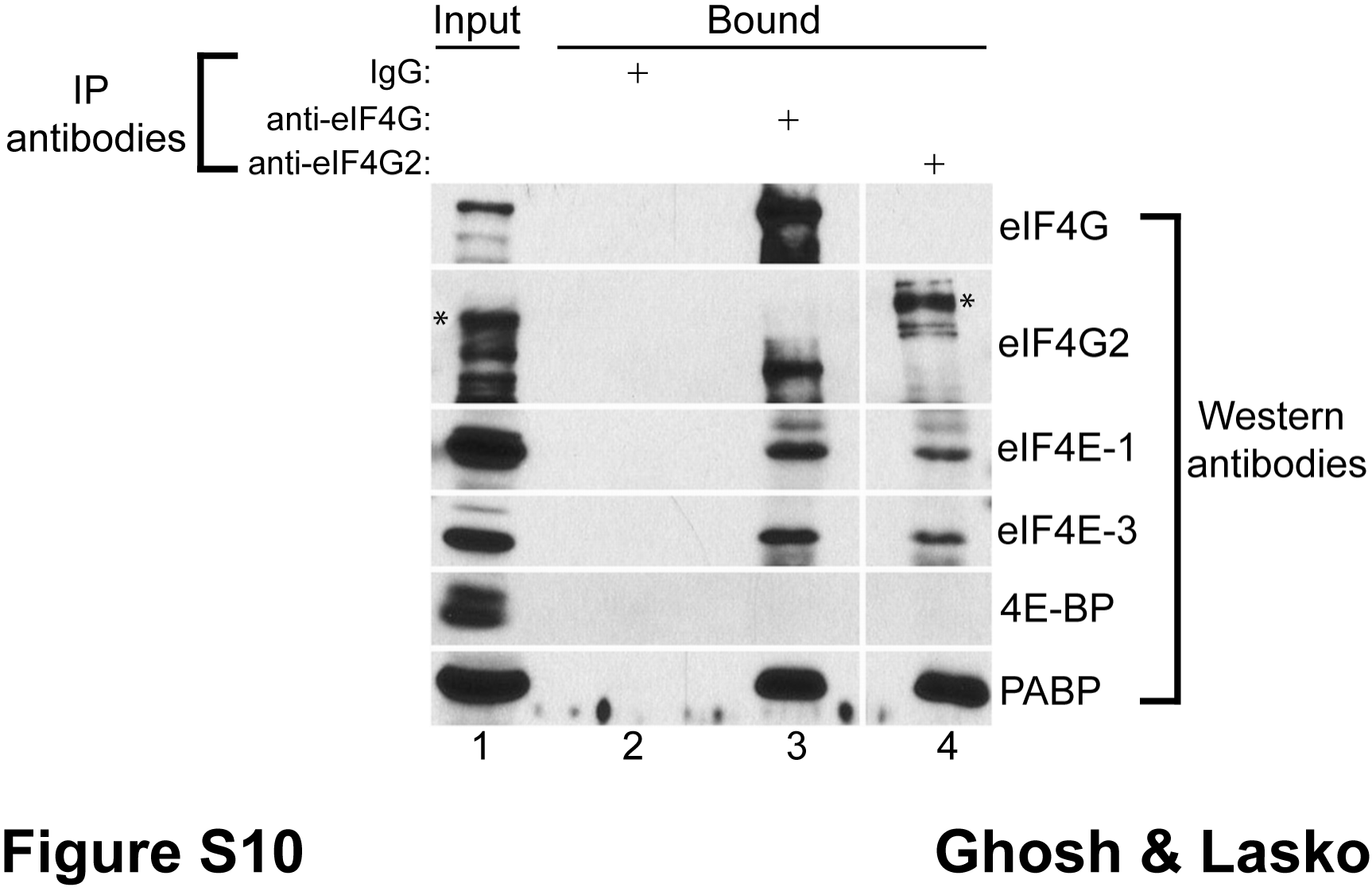

Supplement: S10 Fig — Wild-type testes extract was immunoprecipitated using IgG, anti-eIF4G and anti-eIF4G2 antibodies. The bound proteins (lanes 2, 3, 4) were western blotted and stained with the antibodies indicated at the right of the panel. The band corresponding to eIF4G2 is marked with an asterisk. Input (5%) is shown in lane 1. (TIF) [file pone.0122519.s010.tif]
